# Sustainable oxygen evolution electrocatalysis in aqueous 1 M H_2_SO_4_ with earth abundant nanostructured Co_3_O_4_

**DOI:** 10.1038/s41467-022-32024-6

**Published:** 2022-07-27

**Authors:** Jiahao Yu, Felipe A. Garcés-Pineda, Jesús González-Cobos, Marina Peña-Díaz, Celia Rogero, Sixto Giménez, Maria Chiara Spadaro, Jordi Arbiol, Sara Barja, José Ramón Galán-Mascarós

**Affiliations:** 1 Institute of Chemical Research of Catalonia (ICIQ), The Barcelona Institute of Science and Technology (BIST), Avenida Països Catalans 16, 43007 Tarragona, Spain; 2 Departament de Química Física i Inorgànica, Universitat Rovira i Virgili, Marcel. lí Domingo 1, 43007 Tarragona, Spain; 3 Centro de Física de Materiales, CFM/MPC, (UPV/EHU-CSIC), 20018 San Sebastián, Spain; 4 Institute of Advanced Materials (INAM), Universitat Jaume I, 12006 Castelló, Spain; 5 Catalan Institute of Nanoscience and Nanotechnology (ICN2), CSIC and BIST, Campus UAB, Bellaterra, 08193 Barcelona, Catalonia Spain; 6 ICREA, Passeig Lluis Companys, 23, 08010 Barcelona, Spain; 7 Departamento de Polímeros y Materiales Avanzados: Física, Química y Tecnología, Centro de Física de Materiales, University of the Basque Country UPV/EHU, 20018 San Sebastián, Spain; 8 Donostia International Physics Center, 20018 San Sebastián, Spain; 9 Present Address: Institut de Recherches sur la Catalyse et l’Environnement de Lyon, UMR 5256, CNRS, Université Claude Bernard Lyon 1, 2 Avenue A. Einstein, 69626 Villeurbanne, France; 10 Present Address: Donostia International Physics Center, 20018 San Sebastián, Spain

**Keywords:** Electrocatalysis, Electrocatalysis, Nanoscale materials

## Abstract

Earth-abundant electrocatalysts for the oxygen evolution reaction (OER) able to work in acidic working conditions are elusive. While many first-row transition metal oxides are competitive in alkaline media, most of them just dissolve or become inactive at high proton concentrations where hydrogen evolution is preferred. Only noble-metal catalysts, such as IrO_2_, are fast and stable enough in acidic media. Herein, we report the excellent activity and long-term stability of Co_3_O_4_-based anodes in 1 M H_2_SO_4_ (pH 0.1) when processed in a partially hydrophobic carbon-based protecting matrix. These Co_3_O_4_@C composites reliably drive O_2_ evolution a 10 mA cm^–2^ current density for >40 h without appearance of performance fatigue, successfully passing benchmarking protocols without incorporating noble metals. Our strategy opens an alternative venue towards fast, energy efficient acid-media water oxidation electrodes.

## Abstract (web summary)

While water electrolysis offers a renewable way to produce hydrogen, there are few Earth-abundant, acid-stable water oxidation catalysts. Here, authors show Co_3_O_4_, when protected by a partially hydrophobic environment, to sustain 40 h activity without structural or chemical transformations.

## Introduction

Hydrogen is considered the most environmentally friendly alternative fuel to replace traditional fossil energy^1,2^. However, hydrogen production is still dominated worldwide by natural gas steam reforming, a mature technology fixing a very low competitive price in the market. Electrochemical water splitting powered by renewable sources is regarded as the ideal future technology to produce hydrogen, but costs must be lowered to improve its market penetration^3,4^. The catalysts responsible to improve the efficiency of the process, mainly relying on noble metals, are part of the high cost of the technology, and because of this, many laboratories in the world are working to find viable solutions to develop effective, earth-abundant, robust and scalable catalyst^5–8^.

Acidic electrolytes offer many advantages for hydrogen production, given that high H^+^ concentration improves the hydrogen evolution reaction (HER), and also facilitates fast/low resistance ionic transport^9,10^. Several electrocatalysts based on low-cost raw materials have shown great promise to substitute the state-of-the-art platinum electrodes responsible for HER^6,7,11–14^. On the contrary, no viable candidates are known to substitute noble-metal oxides such as IrO_2_ at the anode where the oxygen evolution reaction (OER) takes place^1,6,9,15–21^. So far, no stable and inexpensive OER catalysts can work under high potential and/or current densities in acidic media, where even the highly active RuO_2_ presents serious deactivation problems^22,23^.

Several strategies have been proposed to promote OER at high proton concentration^24–28^. One strategy deals with the investigation of ternary/complex oxide structures such as nickel-manganese antimonate. This rutile-type oxide was stable for 168 h at 10 mA cm^–2^ operating in 1 M sulfuric acid, although with the penalty of requiring a large overpotential (*η* ≥ 700 mV)^26^. Cobalt-doped hematite thin-film electrocatalysts were also able to sustain a geometric current density of 10 mA cm^–2^ for up to 50 h at pH 0.1, but again at large overpotentials (*η* ≥ 650 mV)^27^. Indeed, cobalt oxides have shown promising OER electrocatalysis in acidic media, although highly limited by its redox potential-dependent instability, since CoO_*x*_ may rapidly dissolve either at open circuit conditions or under high applied potentials^29–32^. Some interesting advances in long-term stability were achieved by doping or processing techniques^33–35^.

In our previous work with polyoxometalate (POM)-supported catalysts, we disclosed how these molecular catalysts showed promising OER catalytic performance in acidic conditions when incorporating active Co centers^36^. The high activity of these catalysts, even in heterogeneous conditions, was achieved thanks to the synergic stability offered by a partially hydrophobic carbon-based support. However, this strategy was not successful to achieve long-term stability, since these POM-based electrodes could only survive at low current densities and for a very limited time, given their intrinsic instability to the mechanical stress provoked by gas bubbling.

In this work, we present a promising processing protocol, which combines in one single anode two powerful strategies: (i) the incorporation of a nanostructured OER catalyst from earth-abundant metals to maximize active surface area, (ii) supported by a conducting, partially hydrophobic binder made from paraffin oil and graphite powder. Our processing protocol with nitrogen-doped carbon-coated Co_3_O_4_ nanoparticles (Co_3_O_4_@C) delivers robust and scalable anodes that exhibit excellent acidic OER performances, needing a minimum overpotential (*η* ≤ 398 mV) to maintain a 10 mA cm^–2^ current density for >40 h when working in 1 M sulfuric acid solution, without any sign of fatigue or deactivation. The high activity and also great stability demonstrate a performance superior to any other non-noble catalysts reported. Oxygen evolution quantification confirms the Faradaic efficiency (>96%) of these electrodes towards OER, with negligible participation of other oxidation processes. These results open alternative opportunities for stable OER electrocatalysis with earth-abundant raw materials.

## Results and discussion

### Catalyst and electrode preparation

**Fig. 1:**
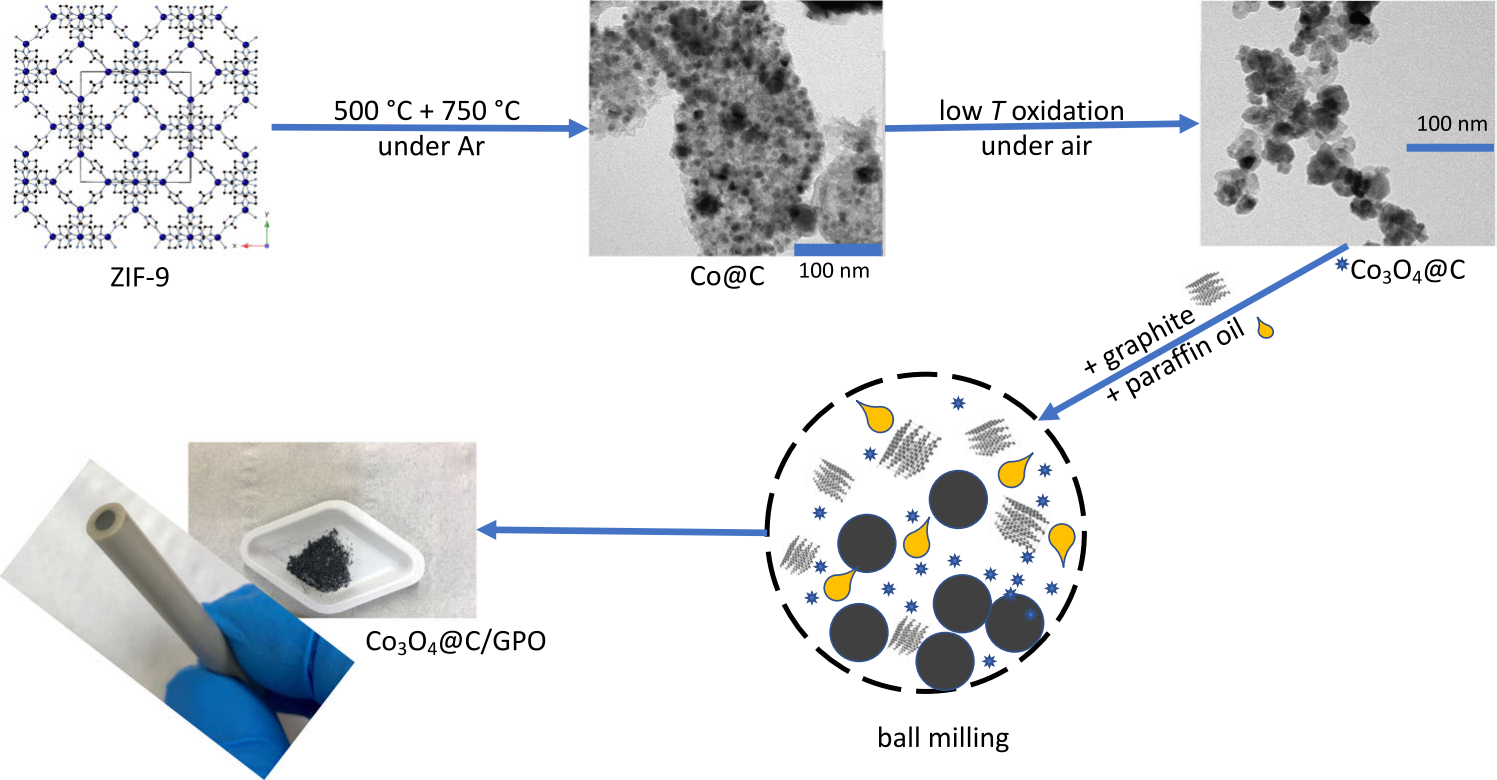
Electrode preparation. Scheme of the synthesis and processing protocol to obtain a Co_3_O_4_@C/GPO electrode.

**Fig. 2:**
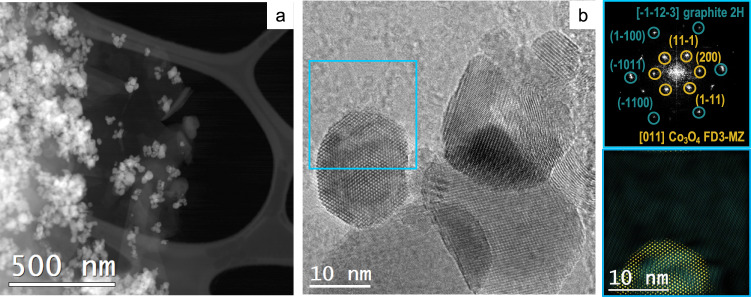
Structural and morphological characterization of Co_3_O_4_@C/GPO composites. HAADF STEM (**a**) and HRTEM (**b**) micrographs from a fresh Co_3_O_4_@C/GPO composite. The power spectrum analysis confirms the presence of Co_3_O_4_ nanoparticles with a cubic FD3-MZ (s.g. 227) spinel structure, oriented along its [011] axis (yellow circles) superimposed to the reflections corresponding to graphite layers with *a* ≈ 0.34 nm spacing and here oriented along the [–1–12–3] (turquoise circles). To highlight the different systems, we show a frequency filtered map (on the bottom right) where the Co_3_O_4_ corresponding lattice fringes are in yellow and the graphite layer ones in turquoise . Source data are provided as a Source Data file.

The overall synthetic and processing protocol is summarized in Fig. 1. We synthesized carbon-coated Co (Co@C) nanoparticles, starting from the thermal treatment of Co(bIm)_2_ (bIm = 2-benzimidazolate), a metal-organic framework (MOF) precursor (ZIF-9)^37^. Then Co@C was oxidized at low-temperature to achieve its full transformation into cobalt oxide nanoparticles, covered by an amorphous, nitrogen-doped-carbon coating derived from the organic skeleton (Co_3_O_4_@C, Fig. 1). Powder X-ray diffraction (PXRD) patterns and Raman spectra confirmed the presence of a Co_3_O_4_ phase and the carbon support (Supplementary Figs. 1–4). High-resolution transmission electron microscopy the presence of graphitic-like nanostructures all around the sample, embedding the Co_3_O_4_ nanoparticles. Some of these C-nanostructures had a nanosheet-like morphology (Fig. 2), while some others were folded forming onion-like rings around the Co_3_O_4_ nanoparticles (Supplementary Fig. 5). Electron energy loss spectroscopy in scanning TEM mode (EELS-STEM) confirmed the chemical composition of the nanoparticles and surrounding nanostructures (Supplementary Fig. 6). The Co_3_O_4_@C composition was determined as (Co_3_O_4_)(H_2_O)_0.30_(OH)_0.85_C_2.00_N_0.05_ by thermogravimetry elemental analysis (Supplementary Fig. 7 and Supplementary Table 1).

For the preparation of the electrode composites, Co_3_O_4_@C was mixed with graphite (G) and paraffin oil (PO) in the desired ratio (see Methods section) to prepare a homogeneous composite (Co_3_O_4_@C/GPO) with the desired Co_3_O_4_@C content up to 40% (40-Co_3_O_4_@C). Composites above 40% were mechanically too fragile for further processing into the working electrode pocket. HRTEM images and EELS-STEM maps showed similar nanostructures within Co_3_O_4_@C/GPO and close contact between Co_3_O_4_@C and GPO (Fig. 2 and Supplementary Fig. 8). X-ray photoemission spectroscopy (XPS) analysis was employed to further identify the surface chemical composition and the mixed oxidation state Co^2+/3+^ (Supplementary Fig. 9) consistent with the presence of the Co_3_O_4_, as confirmed by PXRD and HR-TEM data^38^. XPS spectra from the Co_3_O_4_@C/GPO composite show no differences respect to the Co_3_O_4_@C precursor, demonstrating the absence of chemical modification during composite preparation.

### OER electrocatalytic activity in 1 M H_2_SO_4_

**Fig. 3:**
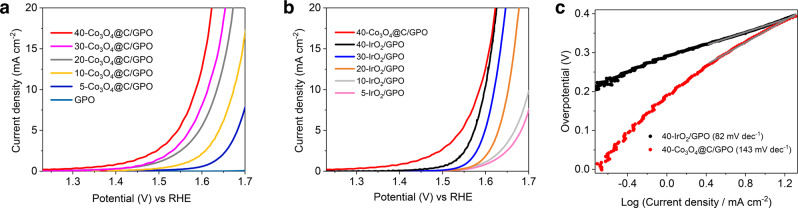
Electrocatalytic activity of Co_3_O_4_@C/GPO electrodes in 1 M H_2_SO_4_ electrolyte. **a** LSV curves for x-Co_3_O_4_@C/GPO electrodes in 1 M H_2_SO_4_ (pH 0.1), at *x* = 0 (GPO blank, light blue), 5 (blue), 10 (yellow), 20 (grey), 30 (pink), 40 (red) and with 1 mV s^–1^ scan rate; **b** LSV curves of *x*-IrO_2_/GPO electrodes in 1 M H_2_SO_4_ (pH 0.1), at *x* = 5 (pink), 10 (gray), 20 (orange), 30 (blue), 40 (black) compared to 40-Co_3_O_4_@C/GPO (red); **c** Tafel plots of IrO_2_/GPO (black) and Co_3_O_4_@C/GPO (red) extracted from LSV data. Source data are provided as a Source Data file.

The *x*-Co_3_O_4_@C/GPO composites (*x* corresponds to the % in weight for the metal oxide) were inserted into the pocket of a working electrode and used as anode during electrochemical water oxidation in 1 M H_2_SO_4_ (pH ≈ 0.1). The cyclic voltammetry (CV) showed the appearance of a catalytic current density on the Co_3_O_4_@C/GPO electrode at relatively low overpotentials, which was sustained after successive cycling curves (Supplementary Figs. 10 and 11). Comparative linear sweep voltammetry (LSV) showed an enhanced electrochemical activity upon increasing Co_3_O_4_@C content, reaching a very low onset overpotential (*η*_onset_ = 194 ± 4 mV) for the 40-Co_3_O_4_@C/GPO electrode (Fig. 3 and Supplementary Fig. 12). These electrodes reach 10 mA cm^–2^ currents at just 360 ± 4 mV overpotential. Interestingly, no sign of a transport-limited regime appeared in the studied potential range, reaching over 20 mA cm^–2^ at *η* = 397 ± 4 mV. A current density limit of 100 mA cm^−2^ was found in these *x*-Co_3_O_4_@C/GPO electrodes.

We prepared analogous IrO_2_/GPO working electrodes to benchmark our results in the same conditions with the state-of-the-art IrO_2_. The IrO_2_/GPO anodes delivered higher overpotentials, ${{{{{{\rm{\eta }}}}}}}_{{{{{{\rm{j}}}}}}=10{{{{{\rm{mA}}}}}}{{{{{{\rm{cm}}}}}}}^{-2}}=368\,{{{{{\rm{mV}}}}}}$ at 10 mA cm^–2^ and ${{{{{{\rm{\eta }}}}}}}_{{{{{{\rm{j}}}}}}=20{{{{{\rm{mA}}}}}}{{{{{{\rm{cm}}}}}}}^{-2}}=396\,{{{{{\rm{mV}}}}}}$ at 20 mA cm^–2^, slightly above those obtained for the Co_3_O_4_@C-based electrode (Supplementary Table 2).

Tafel analyses of the LSV data yielded slopes of 143 mV dec^–1^ for Co_3_O_4_@C and 82 mV dec^–1^ for IrO_2_ (Fig. 3c), suggesting a different reaction mechanism (rate-limiting step) for these two catalysts, and indicating a faster increment of current density with the applied potential for IrO_2_^39,40^. Interestingly, this is compensated by the lower onset potential of Co_3_O_4_@C/GPO. The electrochemical double-layer capacitance (EDLC) of Co_3_O_4_@C/GPO and IrO_2_/GPO were calculated as 25 and 2 mF cm^–2^, respectively, with 0.03 mF cm^–2^ for the blank GPO (Supplementary Fig. 13). This indicates a greater electrochemical active surface area for Co_3_O_4_@C/GPO, due to its higher density of active sites in Co_3_O_4_@C/GPO, thanks to its nanostructuration, favouring the higher current densities observed in the potential range studied^41,42^. It is important to point out that nanostructured IrO_2_ can perform significantly better than our IrO_2_/GPO electrodes^20,43,44^. This must be related to the relatively low electrochemical surface area (ECSA) of our IrO_2_/GPO composite. When we normalize current densities vs. ECSA (Supplementary Fig. 14), IrO_2_/GPO electrodes reach higher values at lower potentials. Still, the Co_3_O_4_@C catalyst remains competitive as a non-noble-metal catalysts, and especially when current density is normalized per gram (Supplementary Fig. 14), as a more practical parameter for applications.

Finally, we measured anodic oxygen evolution during chronopotentiometry experiments with Co_3_O_4_@C/GPO electrodes (Supplementary Fig. 15). We found over >96% Faradaic efficiency, confirming that OER is the dominant process at these electrodes’ surface, and confirming no significant oxidation of the carbon-based matrix is taking place in these conditions.

### OER electrocatalytic stability in 1 M H_2_SO_4_

**Fig. 4:**
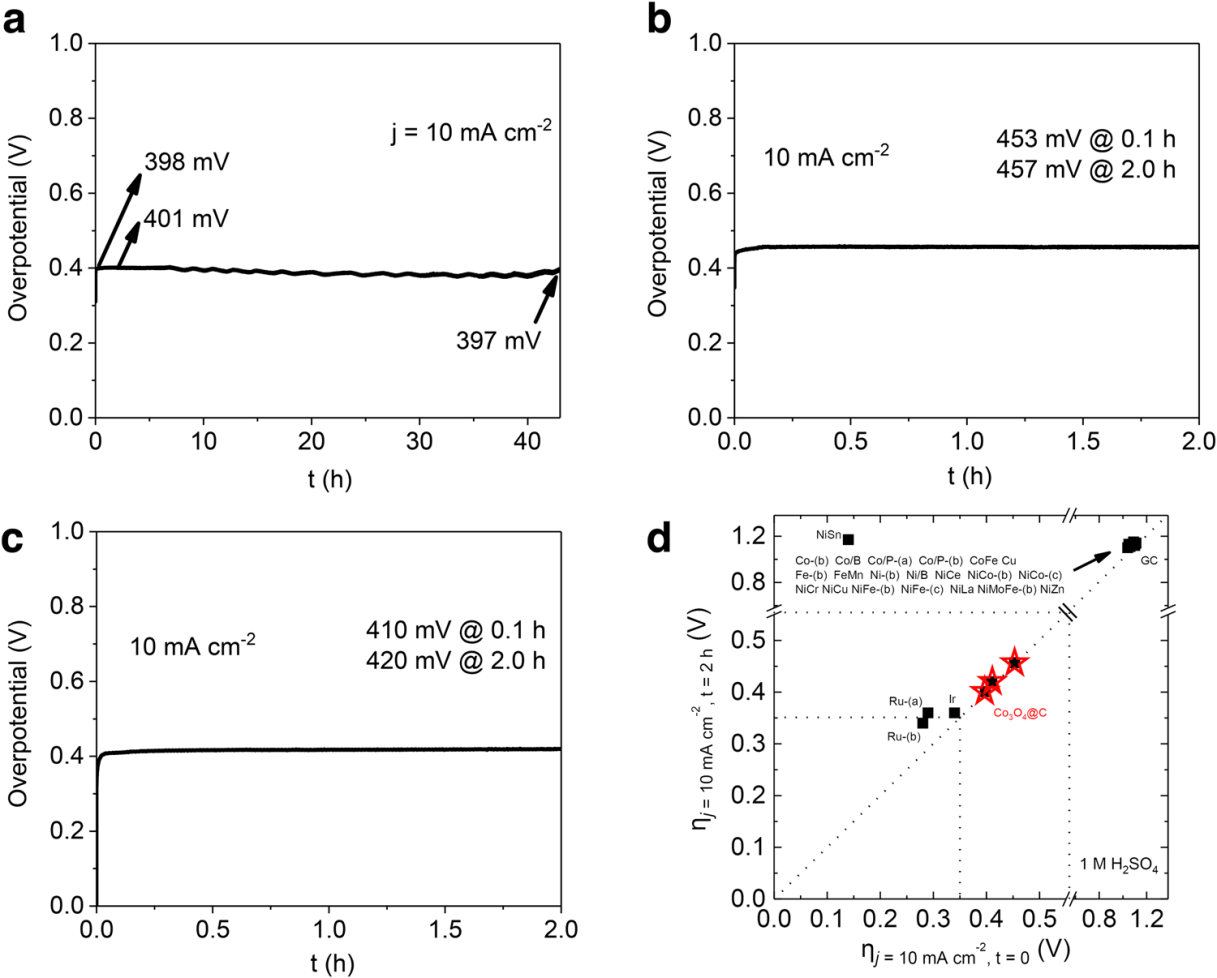
Electrochemical stability of Co_3_O_4_@C/GPO electrodes. Chronopotentiometry measurements at *j* = 10 mA cm^–2^ in 1 M H_2_SO_4_ electrolyte. **a** 40-Co_3_O_4_@C/GPO (>40 h). **b** 20-Co_3_O_4_@C/GPO (2 h). **c** 30-Co_3_O_4_@C/GPO (2 h). **d** Benchmarking of their activity/stability features (red stars) in comparison with other OER electrocatalysts (black squares) in the same electrolysis conditions according to the data from a previous benchmarking study^6^. Source data are provided as a Source Data file.

As mentioned before, stability is a critical issue for earth-abundant OER catalysts in acidic media^45–49^. To determine the stability of our Co_3_O_4_@C/GPO electrodes, we take advantage of the benchmarking protocol designed by Jaramillo’s group that uses as figure of merit the overpotential required to achieve and maintain a 10 mA cm^–2^ current density, as this is approximately the current density expected at the anode in a 10% efficient solar water-splitting device under 1 sun illumination^6,50^. The corresponding chronopotentiometry data (Fig. 4a–c) show very good stability for all electrodes, independently of their Co_3_O_4_@C content. In all cases, ${{{{{{\rm{\eta }}}}}}}_{{{{{{\rm{j}}}}}}=10{{{{{\rm{mA}}}}}}{{{{{{\rm{cm}}}}}}}^{-2}}$ after 2 h shows just a small increment. In the case of our best electrodes, the 40-Co_3_O_4_@C/GPO, this increment is of just 3 mV, and the stability is maintained for long times. After 43 h of continuous electrolysis, the overpotential is essentially identical to the starting value (Fig. 4a). Meanwhile, even to keep 100 mA cm^–2^ current density, only 27 mV increment was needed after 2 h catalysis (Supplementary Fig. 11b). We also applied more aggressive stability tests under cycling applied potentials. In this accelerated degradation testing (ADT), complete CV cycles were collected, thus exposing the electrodes sequentially to catalytic and non-catalytic potentials. These conditions simulate better the different degradation mechanisms during electrode and electrolyzer operation. The 40-Co_3_O_4_@C/GPO electrodes showed also good stability after ~600 CV cycles with only a 14 mV increment in the overpotential to reach  10 mA cm^–2^ current density (Supplementary Fig. 11c).

The benchmarking of these electrodes with previous literature is highlighted in the ${{{{{{\rm{\eta }}}}}}}_{j=10{{{{{\rm{mA}}}}}}{{{{{{\rm{cm}}}}}}}^{-2},t=2{{{{{\rm{h}}}}}}}$ vs. ${{{{{{\rm{\eta }}}}}}}_{j=10{{{{{\rm{mA}}}}}}{{{{{{\rm{cm}}}}}}}^{-2},t=0{{{{{\rm{h}}}}}}}$ plot (Fig. 4d). This comparative plot illustrates the high activity and stability of our electrodes. The three of them appear at the diagonal of the graph, as expected for sustainable performance, and very close and competitive to the results obtained with noble-metal counterparts, indicating that earth-abundant anodes may successfully pass this benchmarking protocol for OER performance in acidic media.

Stability number (S-number) and activity-stability factor (ASF) were also proposed as key metrics for estimating lifetime and long-term stability for electrocatalysts^51–53^. Thus, we analyzed the electrolyte after stability tests to check for Co leaching (Supplementary Table 3). We found the presence of Co but at the ppb level, corresponding to just ≈0.4 % of the total. Based on this number, we can estimate a 25 S-number, an ASF of 101 and a lifetime of 462 h. These estimations are comparable even to Ir-based catalysts such as SrIrO_3_ in analogous conditions, and confirm the promising performance/stability of these electrodes. It is worthy to mention that this small Co loss does not significantly affect performance. This may be due to Co leaching only from non-catalytically areas, or to the 3D nature of our electrodes. Thus, after leaching, the new surface exposed brings additional active sites, keeping the performance. Both may explain why activity is so stable, but a relatively modest S-number is obtained.

### Post-electrolysis Co_3_O_4_@C/GPO characterization

To further confirm the stability of Co_3_O_4_@C as a genuine OER catalyst, we characterized the structural and chemical evolution of the electrodes after these 2 h electrolysis at 10 mA cm^–2^ in 1 M H_2_SO_4_. The powder XRD patterns did not show any significant change nor shift in the observed peaks, still typical of Co_3_O_4_@C and graphite (Supplementary Fig. 16). This suggests no major structural changes are occurring to the bulk of the material Co_3_O_4_@C.

**Fig. 5:**
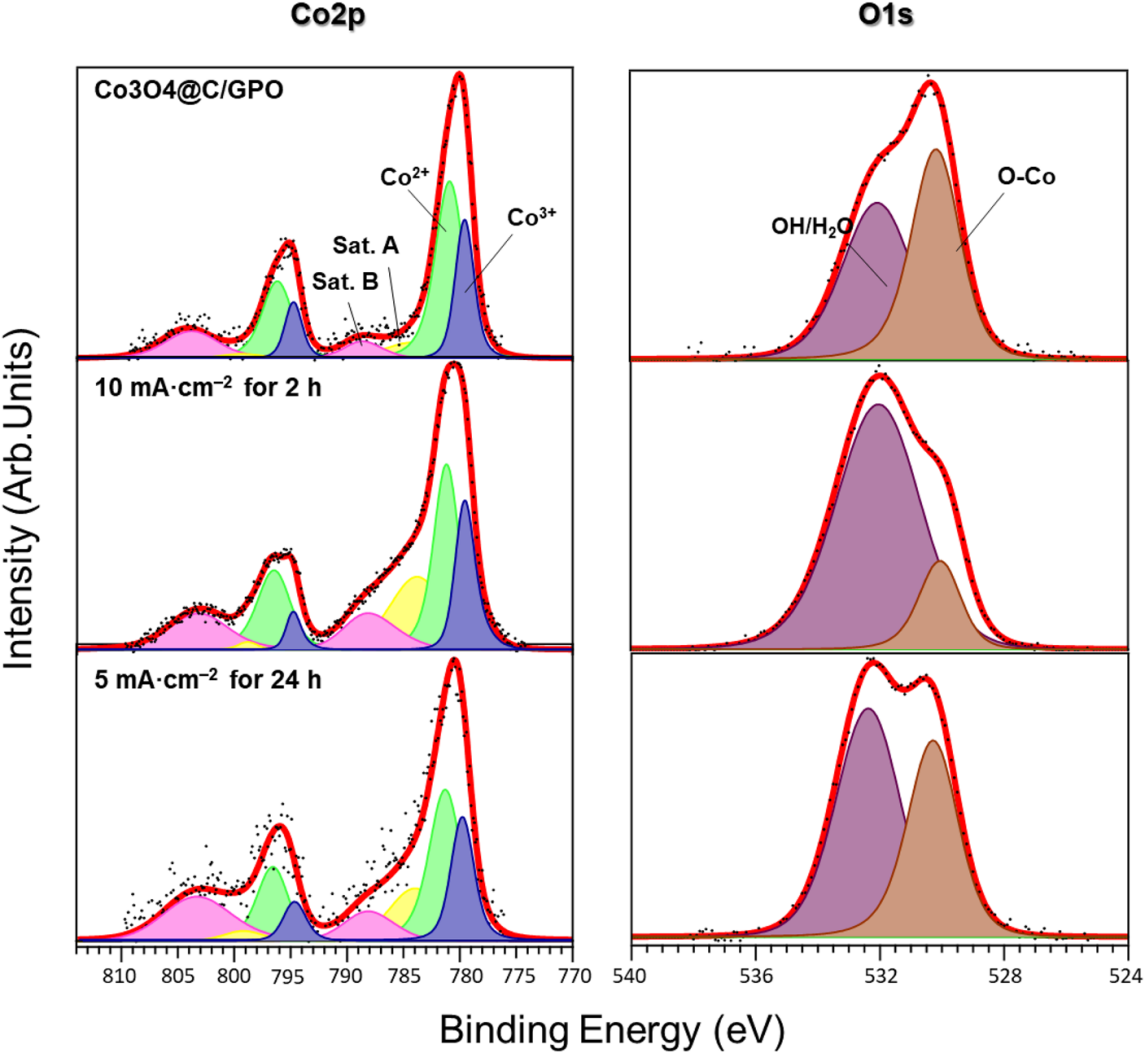
Chemical analysis of the Co_3_O_4_@C/GPO catalyst after water electrolysis. **a** X-ray photoelectron spectra of the Co 2*p* (left) and O 1*s* (right) core levels for a 20-Co_3_O_4_@C/GPO electrode. **b** Same spectra for a 20-Co_3_O_4_@C/GPO electrode after OER chronopotentiometry at 10 mA cm^–2^ for 2 h. **c** Same spectra for a 20-Co_3_O_4_@C/GPO electrode after OER chronopotentiometry at 5 mA cm^–2^ for 24 h. Color assignment for the different areas: Co^3+^ (blue), Co^3+^ satellite (pink), Co^2+^ (green), Co^2+^ satellite (yellow), OH/H_2_O (purple), O–Co (brown). Source data are provided as a Source Data file.

We explored potential changes on the chemical composition of the catalyst due to OER process by XPS characterization of the fresh electrode and after water electrolysis under different conditions. The Co 2*p* XPS spectrum of the fresh Co_3_O_4_@C/GPO electrode (Fig. 5a) shows two peaks located at 794.9 eV (Co 2*p*_1/2_) and 779.7 eV (Co 2*p*_3/2_), corresponding to the spin–orbit splitting of the 2*p* orbital. Both components contain equivalent chemical information. The deconvoluted analysis of the peaks reveals the presence of two different chemical components, which we attribute to the Co^3+^ (blue) and Co^2+^ (green) states, in agreement with the presence of Co_3_O_4_^38^. In addition, we observe two doubled satellite peaks arising from charge transfer and final states effects from Co^2+^ (satellite A, yellow) and Co^3+^ (satellite B, pink)^54^, again characteristic of Co_3_O_4_. We also analyzed the O 1 *s* peak (Fig. 5a). In addition to the Co-O component (brown) related to the Co_3_O_4_, we observe a higher binding energy component attributed to residual OH/H_2_O (purple). Quantitative analysis of the Co 2*p* and O 1 *s* core levels of Co_3_O_4_@C/GPO after chronopotentiometry at 10 mA cm^–2^ for 2 h (Fig. 5b) and 5 mA cm^–2^ for 24 h (Fig. 5c) revealed no shifts in the binding energies of the components respect to the fresh sample. Crucially, the main spectral features attributed to a Co_3_O_4_@C catalyst remain unaltered after water electrolysis, which demonstrates the preservation of the oxidation state of the catalyst. Changes in the intensity of the OH/H_2_O component on the O 1*s* can be fairly attributed to the different environmental conditions of the emersed electrode (see Methods section). XPS analysis of the C component does not evidence significant changes in the oxidation state of the C 1*s* peak, supporting the preservation of the carbon-based matrix (Supplementary Fig. 17) in agreement to the obtained Faradaic efficiencies. Nitrogen detection in the system is below our resolution limit; therefore, no discussion is referred to this element. Based on the current analysis, the most important finding is that the oxide film is stable and no cobalt oxide is lost nor further oxidized during the electrolysis process. In summary, XRD and XPS strongly support the bulk and surface stability of Co_3_O_4_@C before and after acidic OER electrocatalysis, and its genuine catalytic activity.

**Fig. 6:**
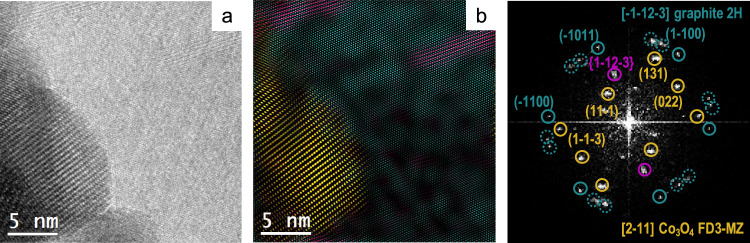
Structural and morphological characterization of Co_3_O_4_@C/GPO electrodes after water electrolysis in 1 M H_2_SO_4_ electrolyte. HRTEM micrograph (**a**) from a Co_3_O_4_@C/GPO composite, recovered after 2 h water electrolysis at 10 mA cm^–2^. To highlight the different systems, we show a frequency filtered map (**b**) where the Co_3_O_4_ nanoparticle lattice fringes are in yellow and the ones corresponding to the surrounding graphitic layers in pink and turquoise. The power spectra (FFT) confirm the high stability of these electrodes, showing again Co_3_O_4_ nanoparticles with a cubic FD3-MZ (s.g. 227) spinel structure (yellow cirlces) here oriented along the [2–11] zone axis. No changes in crystallinity or particle size are observed for the Co_3_O_4_@C composite when compared to the fresh samples (Fig. 2). Notice that the graphitic layers may show multiple rotated domains as shown by the highlighted dotted green circles in the power spectrum, but this effect also happened in the as-prepared sample. Source data are provided as a Source Data file.

We also investigated the Co_3_O_4_@C/GPO composite after 2 h electrolysis at 10 mA cm^–2^ by means of HR-TEM (Fig. 6). The images and power spectra (FFT) analyses also confirm a high structural and chemical stability. Neither crystallinity nor particle size are affected by the electrochemical process.

### Critical role of GPO

To investigate the actual role of the carbon paste in the stability of the electrodes, we carried out additional alternative experiments. First, we directly deposited Co_3_O_4_@C on a glassy carbon (GC) electrode as a Nafion-based ink. This electrode showed a significantly lower electrocatalytic activity when compared with the Co_3_O_4_@C/GPO (Figs. 3–4 vs. Supplementary Fig. 18). More importantly, after 30-min of the benchmarking test in 1 M H_2_SO_4_, the Co_3_O_4_@C/GC electrode is apparently deactivated. This suggests that the GPO binder is fundamental to confer the acidic stability and activity of the Co_3_O_4_@C component.

This effect of the GPO binder could be due to a modified local pH at the electrode/electrolyte interface^55–57^. To check this hypothesis, we decided to investigate the effect of the GPO binder on the local pH through the reversible H^+^/H_2_ pair as catalyzed with commercial Pt/C^58^. The reversible potential for this model reaction differs when the Pt/C is directly deposited on a graphite electrode, or when incorporated into a GPO electrode as observed in their CV plots in a hydrogen saturated 1 M H_2_SO_4_ electrolyte (Supplementary Fig. 19). An average value of –0.002 V vs. *E*_RHE_ was estimated for the Pt/C catalyst, in good agreement with the theoretical +0.0 V value. A + 0.031 V vs. *E*_RHE_ was found for the (Pt/C)/GPO electrode. If we associate this potential difference only to a theoretical local pH difference, ∆*E* = 0.059 ∆pH, we can estimate a pH difference of just 0.52 unit between both electrodes. Of course, this number cannot be assigned to the actual pH at the surface, since this difference may be caused by pH and other parameters, including Pt surface states. However, such small difference indicates that the higher stability and activity found in GPO electrodes cannot arise exclusively from the modification of the local pH. Therefore, we associate the protective function to the hydrophobic environment, which avoids proper solvation of the oxides, precluding its dissolution. The high hydrophobicity of GPO is corroborated by contact angle tests (Supplementary Fig. 20). A contact angle of 125° was found for a 1 M H_2_SO_4_ solution on GPO, significantly higher than on GC (86°).

To prove the general importance of this protective strategy, typical oxides based on Ni, Co, and Fe, which were active in alkaline solution but unstable in acid for oxygen evolution, were synthesized and then their electrochemical performances were measured in 1 M H_2_SO_4_ (Supplementary Fig. 21). It was showed that all of these oxides were active for oxygen evolution and kept their performance at 10 mA cm^–2^ during 2 h catalysis with the small change of ≤13 mV (Supplementary Fig. 22).

We also compared the activity/stability of Co_3_O_4_@C vs. Co_3_O_4_ (Supplementary Fig. 10). The corresponding x-Co_3_O_4_/GPO electrodes showed good stability during preliminary CV cycles and chronopotentiometry measurement, but at higher overpotentials. A 5 mF cm^–2^ EDLC was determined, just 1/5 that of Co_3_O_4_@C/GPO (Supplementary Fig. 13). Specific surface area from N_2_ sorption isotherm curves for Co_3_O_4_@C was also about five times greater than that of Co_3_O_4_ (Supplementary Fig. 23). These results suggest that the role of the carbon coating is to improve the nanostructuration of the active Co_3_O_4_ material.

In addition, the Co_3_O_4_@C/GPO, Co_3_O_4_/GPO, and IrO_2_/GPO electrodes were studied by Electrochemical Impedance Spectroscopy (EIS) at different applied potentials. Supplementary Fig. 24 shows the obtained Nyquist plots, which systematically feature two arcs (or distorted arc for IrO_2_), consistent with two simultaneous/consecutive charge-transfer channels^59^. Fitting the experimental data to a suitable equivalent circuit model (Supplementary Fig. 25) revealed that the best ohmic contact (reflected by the series resistance, R_S_) is obtained for the Co_3_O_4_@C/GPO (Supplementary Fig. 25a). On the other hand, the charge transfer resistance (*R*_ct_), scales inversely with the electrocatalytic activity of the different electrodes, being the lowest one for the Co_3_O_4_@C/GPO electrode (Supplementary Fig. 25b). This is consistent with the estimated surface capacitance, which scales with electrode performance, as a result of higher surface area and hence, higher density of catalytic sites (Supplementary Fig. 25c).

In summary, we are reporting the activity and promising stability of carbon-decorated Co_3_O_4_@C nanoparticles (Co_3_O_4_@C) for electrocatalytic OER under acidic conditions when protected by a hydrophobic binder support. Electrodes built from Co_3_O_4_@C, graphite and paraffin oil are able to evolve oxygen from water during the electrolysis of a concentrated (1 M) sulfuric acid solution (pH < 0.1).

Although previous reports on acidic water splitting with earth-abundant raw materials had achieved either high activity or high stability, our working anodes fulfill both requirements (Supplementary Table 4). These electrodes operate during more than 40 h, at a relatively high current density (10 mA cm^–2^) and at a low overpotential of *η* < 398 mV, very close to the benchmarking performance of state-of-the-art IrO_2_.

The synergy between active catalytic phase, Co_3_O_4_ nanoparticles, and the carbon support (doped-carbon cover, graphite and paraffin oil) is crucial to reach this robust performance. We assign this protective effect to the hydrophobicity of the electrode surface, which could avoid proper solvation of the metal oxides precluding their dissolutions. Indeed, we found preliminary data that supports this approach to be general, also effective when applied to other non-noble-metal-based OER catalysts for working in acid.

Despite the promising performance of our electrodes, a few challenges will need to be addressed before their implementation into commercial electrolyzers. To start with, the carbon content may become an issue at high current densities, as those expected from commercial devices (>500 mA cm^–2^). However, our successful corrosion-protection opens an interesting strategy that can be translated into full cell devices, looking for alternative approaches to incorporate hydrophobic species at the electrode surfaces.

In addition, it is worthy to mention that the current densities reached and sustained by the Co_3_O_4_@C/GPO electrodes are high enough to satisfy the needs of photoelectrochemical (PEC) devices, well in line with the maximum currents provided by photoanodes. Investigations to combine these acid-stable electrodes as co-catalyst for photoactive semi-conductors are under way.

## Methods

### Materials and chemicals

All the chemical reagents and solvents were of commercial grade and used directly without any further purification Experimental Details.

### Synthesis

ZIF-9 was prepared via a solvothermal method according to the previous literature with some modifications^60,61^. Co(NO_3_)_2_·6H_2_O (0.175 g) and benzimidazole (0.142 g) were dissolved into 15 mL DMF and then the homogeneous solution was transferred into a Teflon-lined stainless autoclave. The sealed autoclave was put into an oven and kept at 140 °C for 24 h. When it was cooled down to room temperature, the purple product was filtered out and washed with acetone, and then dried at 60 °C.

To obtain the target material, Co@C was firstly synthesized using ZIF-9 as precursor by heating at 500 and then 750 °C for 2 h, respectively, under Ar flow while Co_3_O_4_ was obtained under air. Afterwards, the pyrolysis product was oxidized in air at 230 °C for 48 h to generate Co_3_O_4_@C.

Mixed metal oxides (NiO_*x*_, FeO_*x*_, NiFeO_*x*_, CoFeO_*x*_, and NiCoO_*x*_) were prepared by modified method available in the literature^62^. Metal nitrates with ratio 1:1 were dissolved in 50 mL of distilled water with constant stirring until a clear solution was obtained. The whole metal concentration was fixed to 0.0125 M. Glycine was added into the aqueous solution (glycine/metal molar ratio is 1.2) and stirred until total dissolution. Afterwards, the solution was heated up to 210 °C until total solvent evaporation and glycine combustion. The resulting porous dark solid was recovered and calcined at 1100 °C in a tubular oven for 1 h.

The composite electrodes were prepared by 2-h ball-milling at 20 s^−1^ of a mixture of paraffin oil (20 mg), graphite powder (80 mg) and the desired weight of metal oxide (5, 10, 20, 30 or 40 mg), namely, x-Co_3_O_4_@C/GPO. x-IrO_2_/GPO (commercial IrO_2_ from AlfaAesar), x-Co_3_O_4_/GPO and other oxide electrodes were also prepared using the same process for comparison purposes.

### Structural characterization

Powder X-ray diffraction (PXRD) data were recorded with a Bruker D8 Advance Series equipped with a VANTEC-1 PSD3 detector. Elemental analyses were carried out with an Agilent 725-ES inductively coupled plasma optical emission spectrometer (ICP-OES) at University of Valladolid (Co) and LECO CHNS-932 elementary microanalyzers (C, H, N) at Complutense University of Madrid. Thermogravimetric analysis was conducted with a thermogravimetric balance of Mettler Toledo. Nitrogen adsorption-desorption isotherms at 77 K were measured on a Quantachrome Autosorb iQ gas adsorption analyzer. Prior to analysis, the sample was degassed in vacuum. The BET method was applied to calculate the total surface area.

### Electrochemistry

All electrochemical experiments were performed under ambient conditions (≈293 K) with a Bio-Logic VMP3 multichannel potentiostat and implemented with a three-electrode configuration using 1 M H_2_SO_4_ (pH 0.1) as filling solution, Pt mesh as counter electrode, Ag/AgCl (3 M KCl) as reference electrode and a pocket working electrode (0.07 cm^2^ surface area and 4 mm depth) filled with the GPO composites. The actual mass amounts of the *x*-Co_3_O_4_@C/GPO composites in the electrode pocket were measured with a weight balance and are indicated in Supplementary Table 5. Although it is difficult to estimate the actual active layer, an estimation is suggested that 1/8 of the total electrode pocket volume is used as the maximum limit in contact with the solution in order to determine the mass loading for comparison (Supplementary Table 5)^36^. All potentials were measured vs. Ag/AgCl electrode and converted to the RHE reference scale using *E*_RHE_ = *E*_Ag/AgCl_ + 0.21 + 0.059 pH (V) while overpotentials *η* = *E*_RHE_ – 1.229 V. All current densities were calculated based on the geometrical surface area of the electrodes. Ohmic drop (R) was determined by using the automatic current interrupt (CI) software and the corresponding ohmic drops were included in Supplementary Table 6. iR-compensations were applied to all electrochemical data. CV experiments were carried out with 10 or 100 mV s^–1^ scan rates. Single LSV curve was recorded with a 1 mV s^–1^ scan rate for activity comparison after 10-CV cycle activation in the 0–1.4 V vs. Ag/AgCl potential range. Tafel slopes were estimated from the LSV curves by plotting overpotential *η* vs. log *j* (*j* = current density). The potential vs. RHE to drive 1 mA cm^–2^ was used to define onset potential and corresponding *η*_onset_. Chronopotentiometry tests were carried out at fixed current densities of 10 or 100 mA cm^–2^. Accelerated degradation testing (ADT) was measured between 0–1.4 V vs. Ag/AgCl using 100 mV s^−1^ scan rate^63^. For the electrochemical double-layer capacitance (EDLC) measurements, open circuit potentials (OCPs) vs. the Ag/AgCl were firstly recorded for 30 min to reach rather stable values. Combined with above CV measurements, the 100 mV potential windows centered at OCPs could be determined and cyclic voltammetries were then carried out under scanning rates of 20, 40, 60, 80, and 100 mV s^–1^. The current density differences between the minimum and maximum values at OCPs vs. the Ag/AgCl and the corresponding scanning rates were plotted to calculate the EDLC value (1/2 of the slope of current density-scan rate plots)^64^. Co_3_O_4_@C and commercial Pt/C from AlfaAesar were also deposited on the glassy carbon (GC, 0.07 cm^−2^) disk electrodes. The inks were prepared by sonicating 10 mg of catalyst, 25 μL Nafion 117 containing solution and 975 μL ethanol aqueous solution (3:1 in volume) for 30 min.

Electrochemical impedance spectroscopy (EIS) was performed by means of a typical three-electrode cell in the frequency range from 100 kHz to 0.1 Hz with 8 points per decade. The AC perturbation was 5 mV. Experimental data were fitted to the selected equivalent circuit model using Zview software (Scriber Associates) for extracting both capacitances and resistances.

### Faradaic efficiency

In order to evaluate the faradaic efficiency towards oxygen production, the chronopotentiometric experiment (Supplementary Fig. 15b) was carried out applying a fixed current (1.4 mA, 2 h) while oxygen concentration in the headspace was in situ measured by using an Unisense sensing system equipped with an oxygen microsensor based on voltage polarization. The experiment was performed under continuous flow conditions by bubbling Ar as carrier gas in both anodic and cathodic compartments. For this purpose, an H-type cell was used containing a frit glass separating both compartments, a connection for the sensor to be inserted in the anodic gas headspace, and connections for the inlet and outlet Ar streams in both compartments (Supplementary Fig. 26). The oxygen microsensor was in situ two-point calibrated by feeding to the H-cell with certified standard of Ar (≥99.999%) and compressed air, being the gas flow rate controlled by a set of mass flowmeters (Bronkhorst EL-FLOW).

After purging the cell headspace with argon, the chronopotentiometry test was started and the oxygen concentration was monitored until reaching stabilization. The expected faradaic oxygen production rate (${F}_{{{{{{{\mathrm{O}}}}}}}_{2},{{{{{{{\mathrm{far}}}}}}}}}$ in mol s^−1^) is calculated with the following equation:

1 $${F}_{{{{{{{\mathrm{O}}}}}}}_{2},{{{{{{{\mathrm{far}}}}}}}}}=I\,{n}_{{{{{{\mathrm{e}}}}}}}^{{-}1}{F}^{{-}1}$$

where *I* is the applied current (in A), *n*_e_ is the number of mols of electrons involved in the water oxidation reaction to generate one mol of oxygen (4) and *F* is the Faraday constant (96,485 C mol^–1^).

The experimental O_2_ flow rate (${F}_{{O}_{2},{\exp }}$ in mol s^–1^) was calculated considering ideal gas behavior with the following equation:

2 $${F}_{{O}_{2},{\exp }}=P\,{C}_{{O}_{2}}\,{F}_{{{{{{{\mathrm{Ar}}}}}}}}\,(100-C_{O_{2}})^{{-}1}\,{R}^{{-}1}\,{T}^{{-}1}$$

where *P* is the total gas pressure (1 atm), *C* (O_2_) is the steady-state oxygen concentration provided by the sensor (in %), *F*_Ar_ is the Ar carrier flow (in L s^–1^), *R* is the ideal gas constant (0.082 atm L K^–1^ mol^–1^) and *T* is the cell temperature (293 K).

Then Faradaic efficiency (in %), FE, is calculated as follows:

3 $${{{{{{\mathrm{FE}}}}}}}=\,\frac{100\,{\times F}_{{O}_{2},{\exp }}}{{F}_{{O}_{2},{f_{{{{{{\mathrm{ar}}}}}}}}}}$$

### X-ray photoemission spectroscopy

X-Ray photoemission (XPS) experiments were performed inside an ultra-high vacuum chamber (base pressure of 10^–10^ mbar) using a Phoibos 100 photoelectron spectrometer equipped with an Al Kα X-ray source (16 mA, 1486.6 V) as the incident photon radiation. XPS spectra of Co 2*p*, O 1*s*, N 1*s*, and C 1*s* core levels were measured for as received samples deposited on top of indium tape. The spectra are well described by the superposition of several Doniach-Sunjic curve-components. The intensities of the XPS core levels were evaluated by the peak areas, after a standard background subtraction according to Shirley procedure^65^. The spin–orbit splitting for every component into the Co-2*p* core level has been set to *D* = 15.2 eV with a branching ratio of 0.5. The metallic cobalt peak, Co 2*p*_3/2_ = 779.7 eV, was used for a final calibration of the spectra^38^. Co_3_O_4_@C/GPO after OER chronopotentiometry at 10 mA cm^–2^ for 2 h at was washed with acetone in order to remove the paraffin oil prior to XPS measurements. As expected, the treatment do not affect the oxidation state of the composite, as evidenced by the measured spectra.

### Transmission electron microscopy

High-resolution transmission electron microscopy (HRTEM) and scanning transmission electron microscopy (STEM) investigations were performed on a field emission gun FEI Tecnai F20 microscope. High-angle annular dark-field (HAADF) STEM was combined with electron energy loss spectroscopy (EELS) in the Tecnai microscope by using a GATAN QUANTUM energy filter in order to obtain compositional maps. STEM-EELS maps were performed using the O K-edge at 532 eV (green), the Co L-edge at 779 eV (red) and C K-edge at 284 eV (blue).

### Reporting summary

Further information on research design is available in the Nature Research Reporting Summary linked to this article.

## Supplementary information

Supplementary Information

Reporting Summary

## Data Availability

All data is available in the main text or in the supplementary materials. Source data are provided with this paper.

## Source data

Source Data
