# Author Correction: Sustainable oxygen evolution electrocatalysis in aqueous 1 M H_2_SO_4_ with earth abundant nanostructured Co_3_O_4_

**DOI:** 10.1038/s41467-022-32399-6

**Published:** 2022-08-10

**Authors:** Jiahao Yu, Felipe A. Garcés-Pineda, Jesús González-Cobos, Marina Peña-Díaz, Celia Rogero, Sixto Giménez, Maria Chiara Spadaro, Jordi Arbiol, Sara Barja, José Ramón Galán-Mascarós

**Affiliations:** 1 Institute of Chemical Research of Catalonia (ICIQ), The Barcelona Institute of Science and Technology (BIST), Avenida Països Catalans 16, 43007 Tarragona, Spain; 2 Departament de Química Física i Inorgànica, Universitat Rovira i Virgili, Marcel. lí Domingo 1, 43007 Tarragona, Spain; 3 Centro de Física de Materiales, CFM/MPC, (UPV/EHU-CSIC), 20018 San Sebastián, Spain; 4 Institute of Advanced Materials (INAM), Universitat Jaume I, 12006 Castelló, Spain; 5 Catalan Institute of Nanoscience and Nanotechnology (ICN2), CSIC and BIST, Campus UAB, Bellaterra, 08193 Barcelona, Catalonia Spain; 6 ICREA, Passeig Lluis Companys, 23, 08010 Barcelona, Spain; 7 Departamento de Polímeros y Materiales Avanzados: Física, Química y Tecnología, Centro de Física de Materiales, University of the Basque Country UPV/EHU, 20018 San Sebastián, Spain; 8 Donostia International Physics Center, 20018 San Sebastián, Spain; 9 Present Address: Institut de Recherches sur la Catalyse et l’Environnement de Lyon, UMR 5256, CNRS, Université Claude Bernard Lyon 1, 2 Avenue A. Einstein, 69626 Villeurbanne, France; 10 Present Address: Donostia International Physics Center, 20018 San Sebastián, Spain

**Keywords:** Electrocatalysis, Electrocatalysis, Nanoscale materials

Correction to: *Nature Communications* 10.1038/s41467-022-32024-6, published online 27 July 2022

The original version of this Article contained an error in Fig. 5, in which x-axis beneath panel c was incorrectly labelled. The correct version of Fig. 5 is:
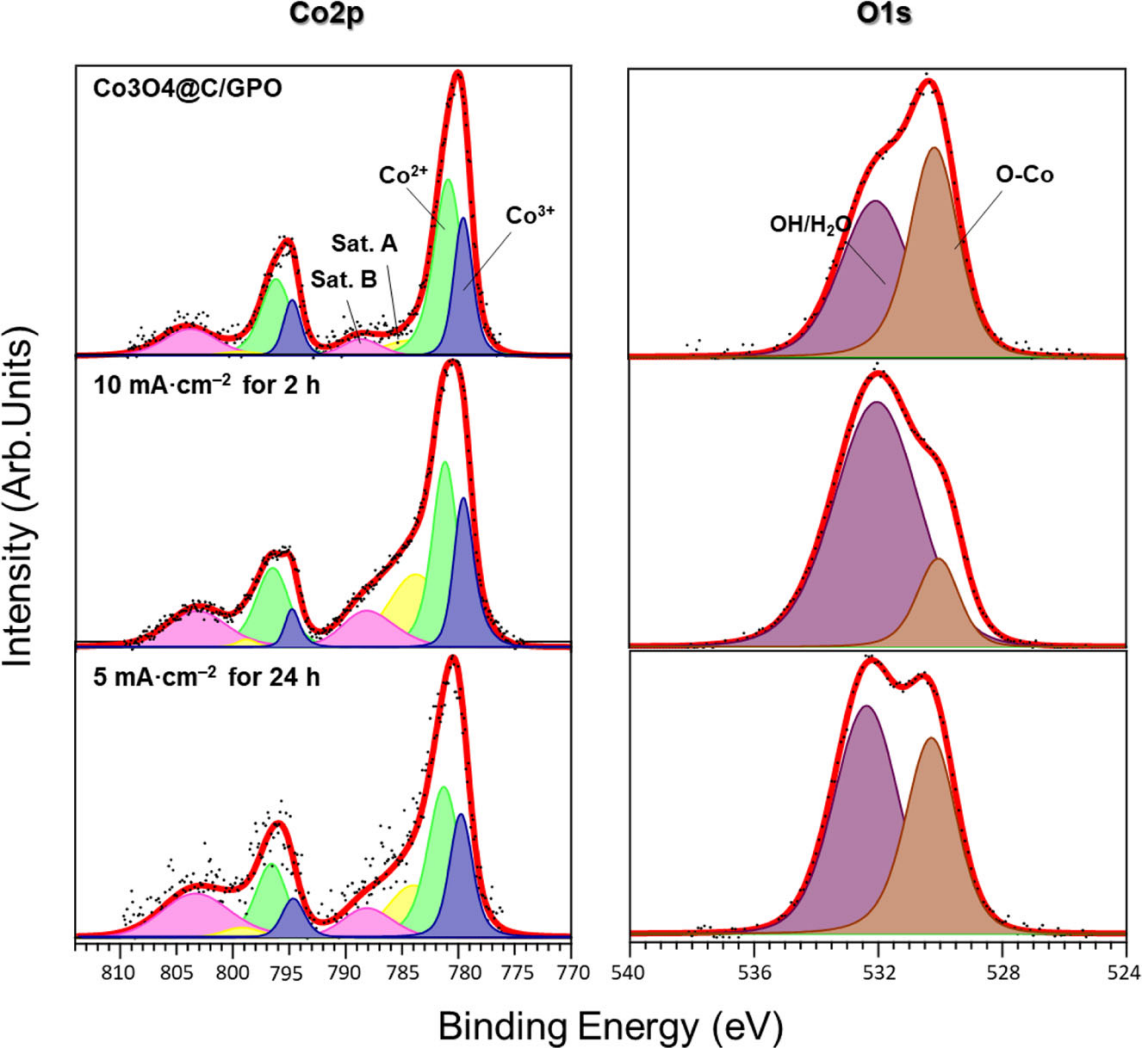


which replaces the previous incorrect version:
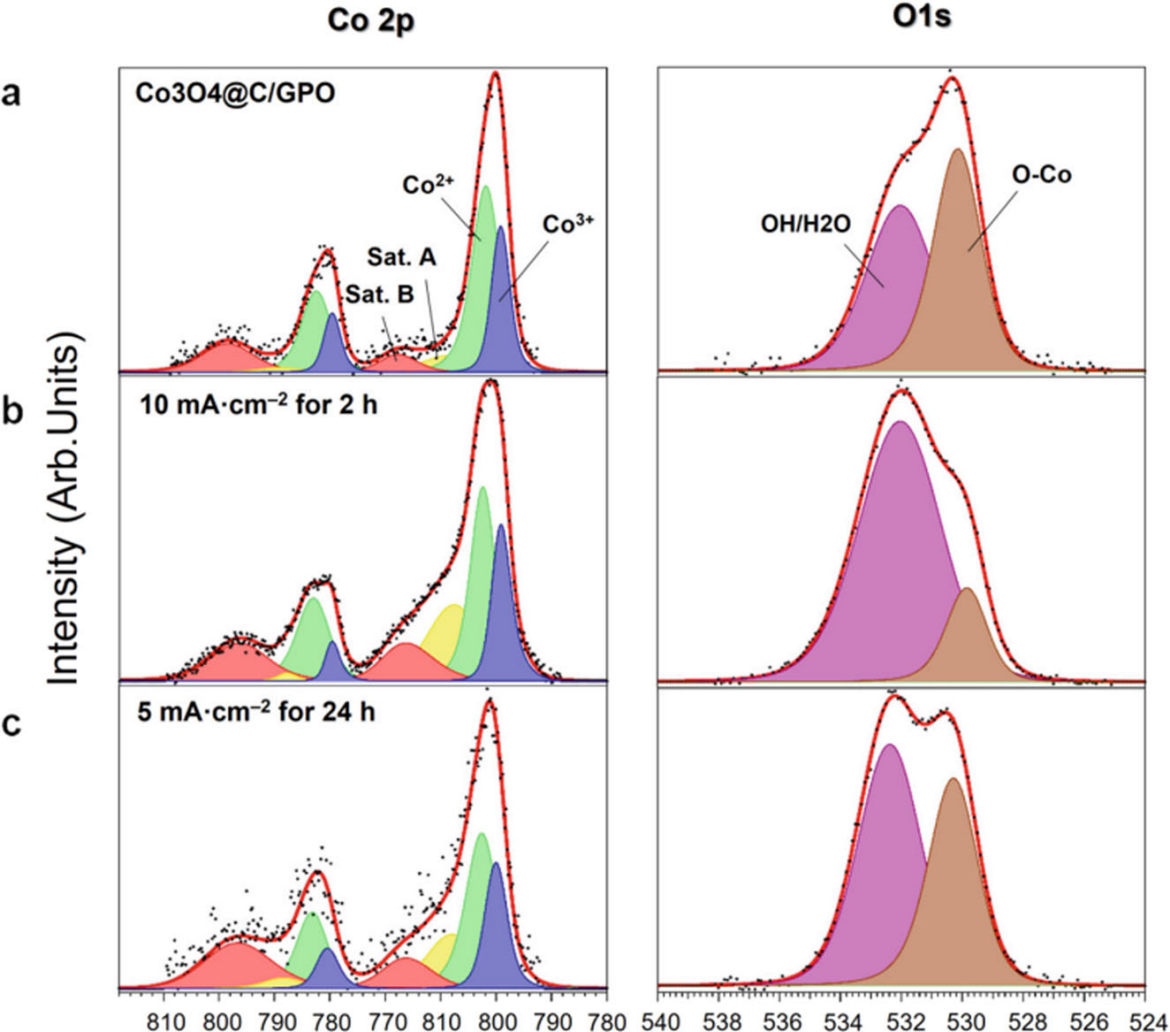


This has been corrected in both the PDF and HTML versions of the Article.

The original version of this Article contained an error in the caption of Fig. 5, in which the colours used are incorrect. The correct version now states “Color assignment for the different areas: Co3+ (blue), Co3+ satellite (pink), Co2+ (green), Co2+ satellite (yellow), OH/H2O (purple), O–Co (brown).” which replaces the incorrect “Color assignment for the different areas: Co3+ (violet), Co3+ satellite (red), Co2+ (green), Co2+ satellite (yellow), OH/H2O (purple), O–Co (brown).” This has been corrected in both the PDF and HTML versions of the Article.

“The original version of this Article contained an error in the second line of the fifth page which incorrectly read ‘B, red)54’. The correct version states ‘B, pink’ in place of ‘B, red.’ This has been corrected in both the PDF and HTML versions of the Article.”

“The original version of this Article contained an error in the fifth line of the fifth page which incorrectly read ‘residual OH/H2O (pink)’. The correct version states ‘(purple)’ in place of ‘(pink)’. This has been corrected in both the PDF and HTML versions of the Article.”

“The original version of this Article contained an error in the fourteenth line of the second column on the seventh page which incorrectly read ‘High-electrochemical impedance spectroscopy’. The correct version states ‘Electrochemical’ in place of ‘High-electrochemical’. This has been corrected in both the PDF and HTML versions of the Article.”

