# Supplementary material for: Sustainable oxygen evolution electrocatalysis in aqueous 1 M H2SO4 with earth abundant nanostructured Co3O4
Source: Nat Commun. 2022 Jul 27;13:4341. doi: 10.1038/s41467-022-32024-6 (PMC9329283; doi:10.1038/s41467-022-32024-6)
Supplement: Supplementary file 1 — Supplementary Information [file 41467_2022_32024_MOESM1_ESM.pdf]

Supplementary Information for

Sustainable oxygen evolution electrocatalysis in aqueous 1 M H_2SO_4 with earth abundant nanostructured Co_3O_4

Jiahao Yu,[1,2] Felipe A. Garcés-Pineda,[1] Jesús González-Cobos,[1,9] Marina Peña-Díaz,[3] Celia Rogero,[3] Sixto Giménez,[4] Maria Chiara Spadaro,[5] Jordi Arbiol,[5,6] Sara Barja,[7,8*] and José Ramón Galán-Mascarós[1,6*]

[1]Institute of Chemical Research of Catalonia (ICIQ), The Barcelona Institute of Science and Technology (BIST), Av. Països Catalans 16, 43007 Tarragona, Spain.

[2]Departament de Química Física i Inorgànica, Universitat Rovira i Virgili, Marcel. lí Domingo 1, 43007 Tarragona, Spain.

[3]Centro de Física de Materiales, CFM/MPC(UPV/EHU-CSIC), 20018 San Sebastián, Spain.

[4]Institute of Advanced Materials (INAM), Universitat Jaume I, 12006 Castelló,

[5]Catalan Institute of Nanoscience and Nanotechnology (ICN2), CSIC and BIST, Campus UAB, Bellaterra, 08193 Barcelona, Catalonia, Spain

[6]ICREA, Passeig Lluis Companys, 23, 08010 Barcelona, Spain.

[7]Departamento de Polímeros y Materiales Avanzados: Física, Química y Tecnología, Centro de Física de Materiales, University of the Basque Country UPV/EHU, 20018 San Sebastián, Spain

[8]Donostia International Physics Center, 20018 San Sebastián, Spain.

[9]*Present affiliation: Institut de Recherches sur la Catalyse et l'Environnement de Lyon, UMR 5256, CNRS, Université Claude Bernard Lyon 1, 2 Avenue A. Einstein, 69626 Villeurbanne, France*

*Correspondence to: sara.barja@ehu.eus; jrgalan@iciq.es

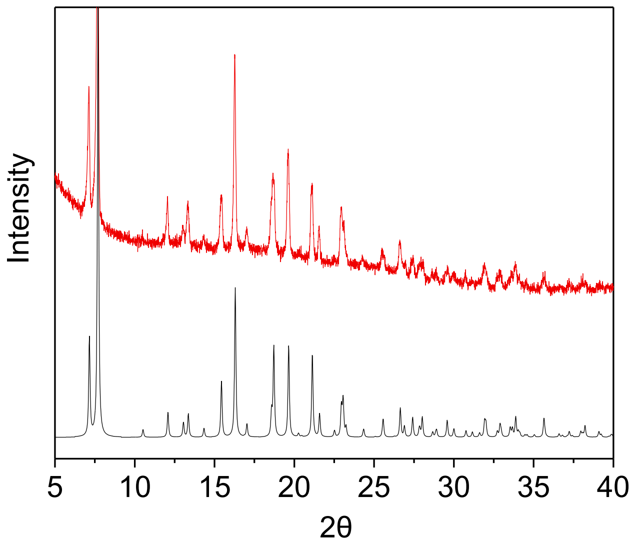

Supplementary Fig. 1. Structural characterization. PXRD patterns of as-synthesized ZIF-9 (red) and the one by Yaghi's group[1] (black).

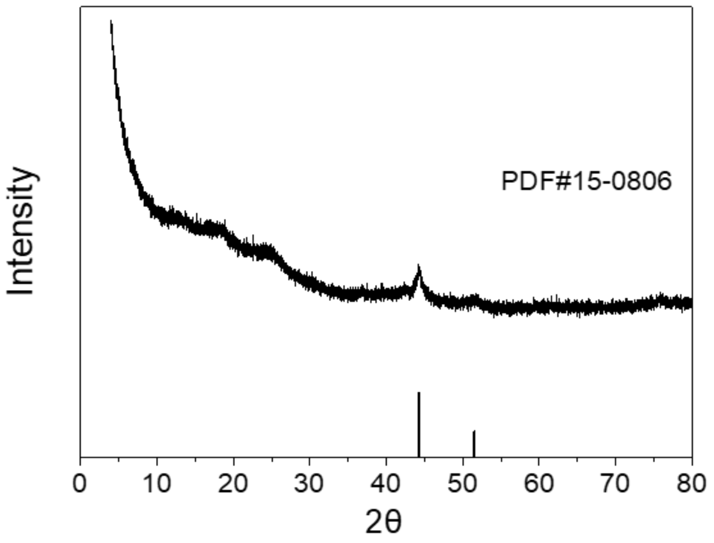

Supplementary Fig. 2. Structural characterization. PXRD pattern for Co@C.

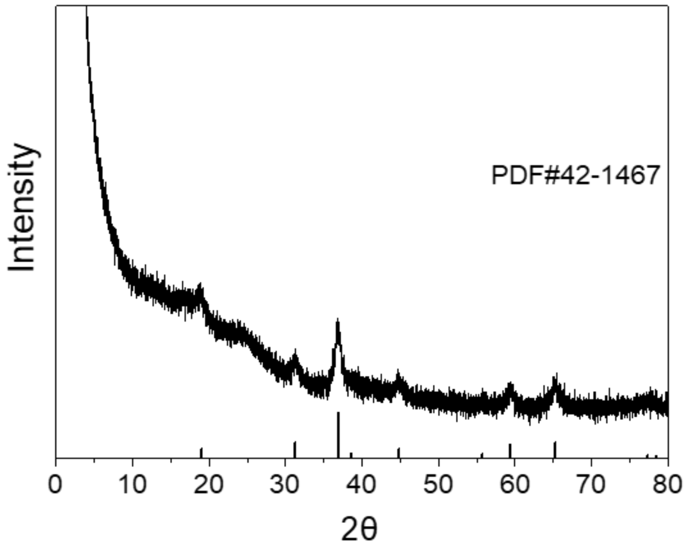

Supplementary Fig. 3. Structural characterization. PXRD pattern for Co_3O_4@C.

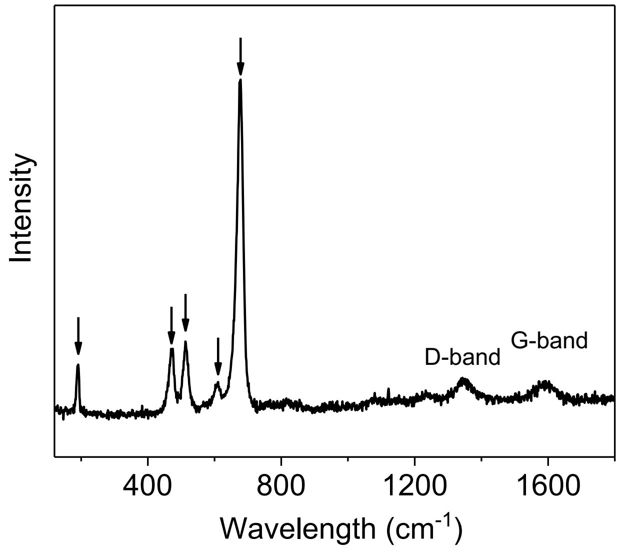

Supplementary Fig. 4. Structural characterization. Raman spectrum for Co_3O_4@C.

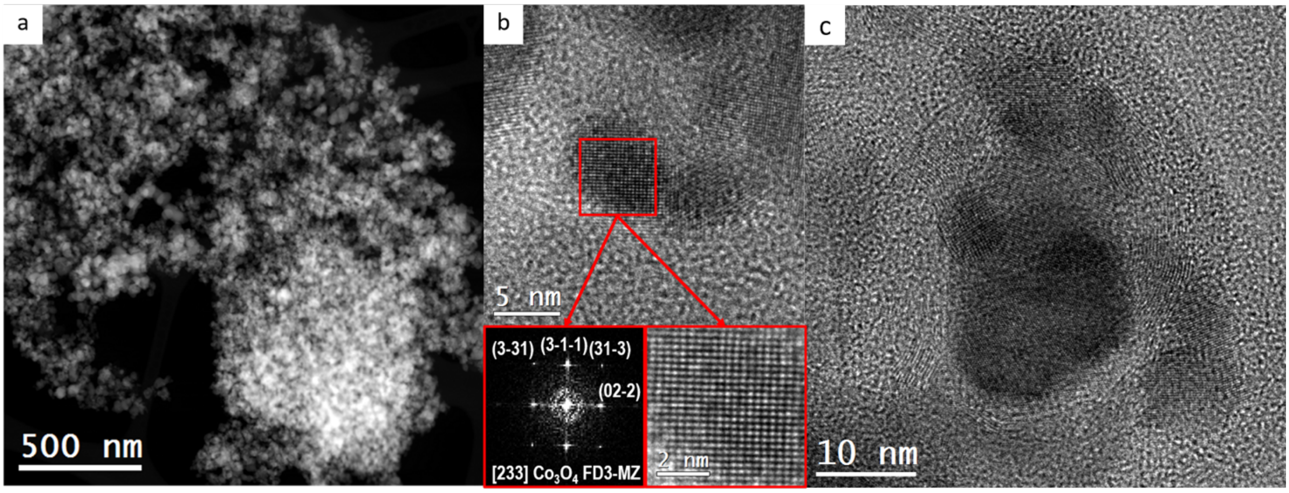

Supplementary Fig. 5. Electron microscopy. Low mag HAADF STEM and HRTEM images for as-prepared Co_3O_4@C together with the power spectrum analysis (a, b, c).

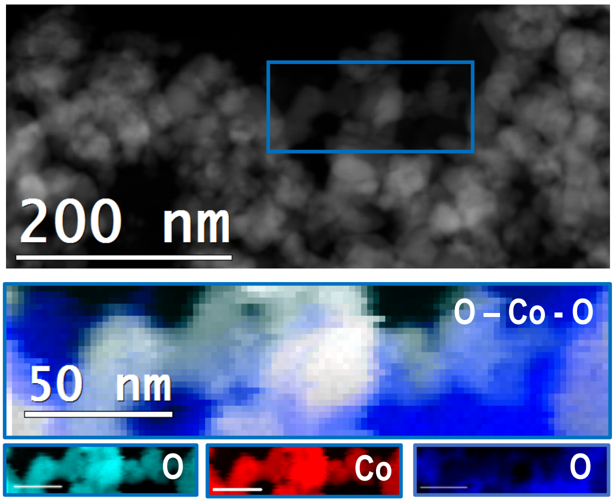

Supplementary Fig. 6. Electron microscopy. STEM-EELS elemental mapping for the as-prepared Co_3O_4@C.

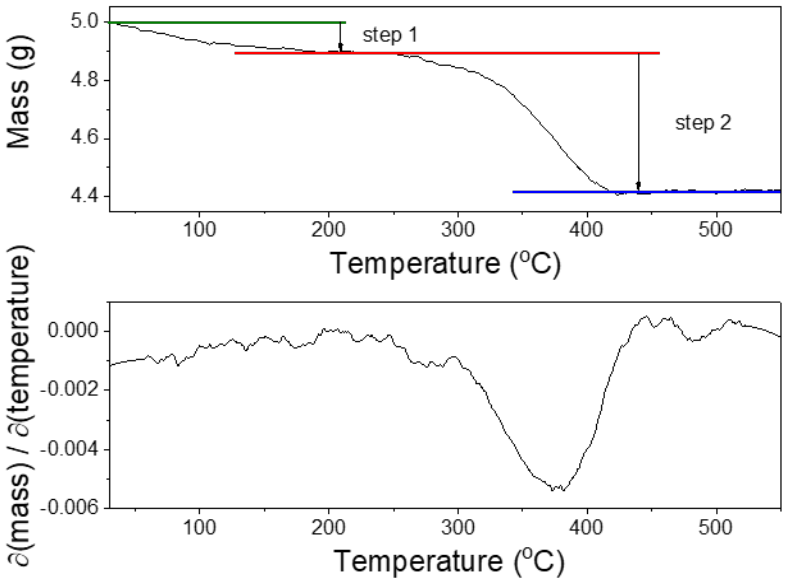

Supplementary Fig. 7. Thermal gravimetry. TGA curves of Co_3O_4@C with the heating rate of 10 °C min^{-1} in air. The step 1 shows the loss of water whose amount is 1.9 wt% while step 2 indicates the loss of carbon, nitrogen or other unstable species and further oxidation of Co_3O_4 to Co_2O_3 at the same time.

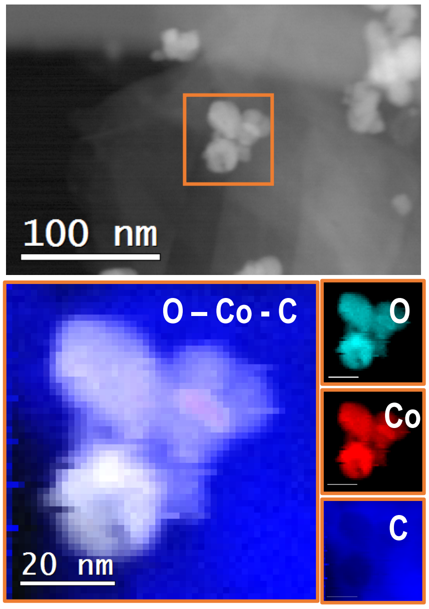

Supplementary Fig. 8. Electron microscopy. STEM-EELS elemental mapping for Co_3O_4@C/GPO.

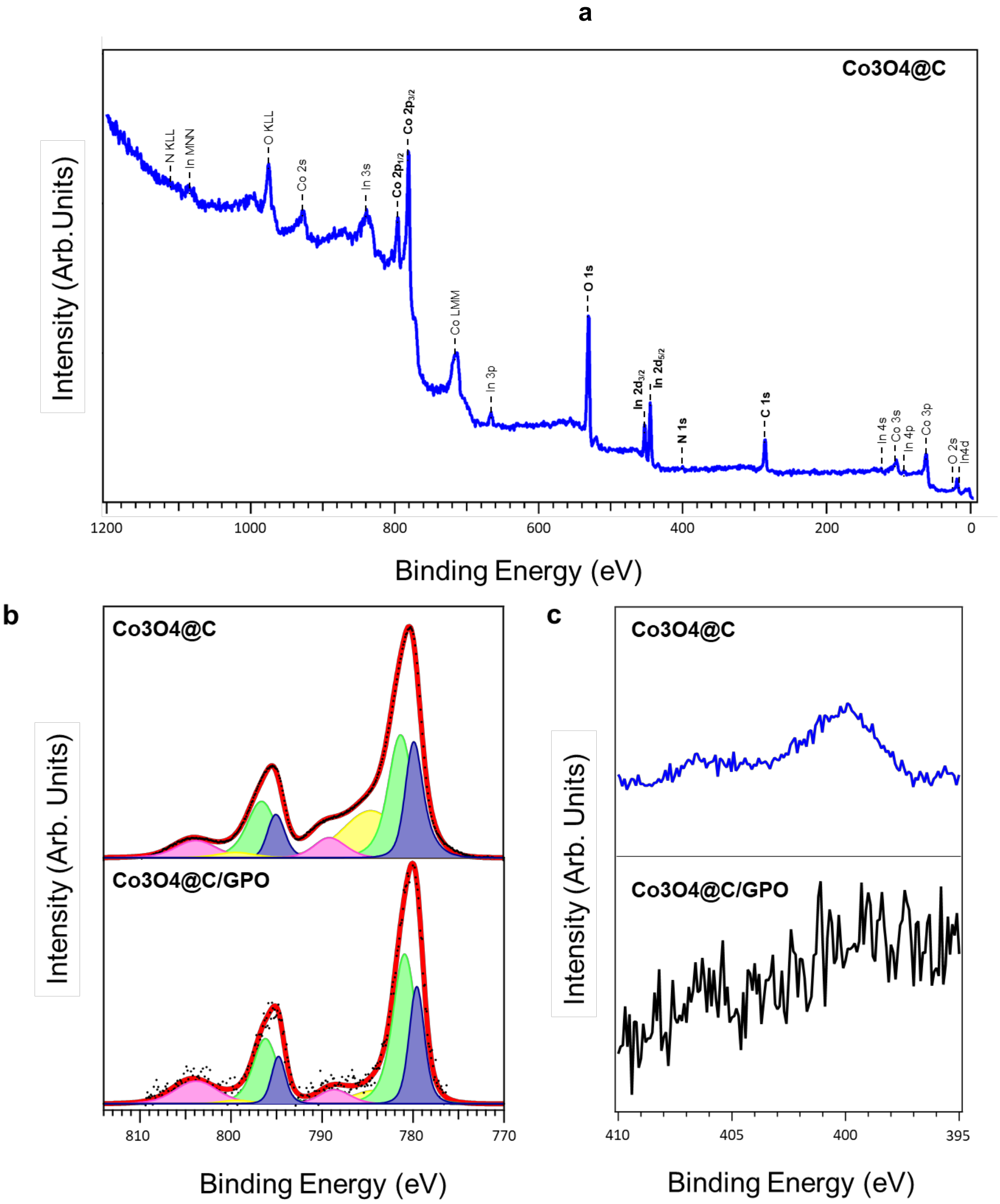

Supplementary Fig. 9. Chemical XPS characterization. (a) Overview spectrum of Co_3O_4@C catalyst with all the relevant core-levels attributed to the electrode composition (Co, O, N and C). No additional elements are identified, supporting the absence of cross-contamination of the catalyst. Indium (In) presence is justified since the samples were deposited onto and In tape for XPS measurement. (b) Co 2p XPS peak of the Co_3O_4@C (upper) and Co_3O_4@C/GPO (bottom) samples. No compositional changes are observed due to composite preparation. (c) N 1s XPS spectra of Co_3O_4@C (upper) and Co_3O_4@C/GPO (bottom) samples. Nitrogen detection in the system is below our resolution limit when the electrode composite is prepared.

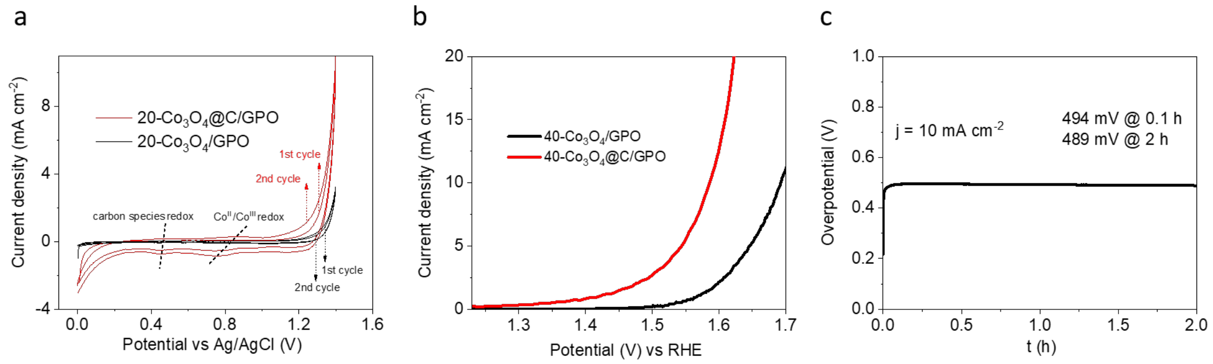

Supplementary Fig. 10. Additional electrochemical data: (a) CV curves of 20-Co_3O_4@C/GPO and 20-Co_3O_4/GPO, respectively. (b) LSV curves of 40-Co_3O_4@C/GPO and 40-Co_3O_4/GPO in 1 M H_2SO_4 electrolyte and (c) stability in chronopotentiometry measurements at 10 mA cm^{-2} of 40-Co_3O_4/GPO.

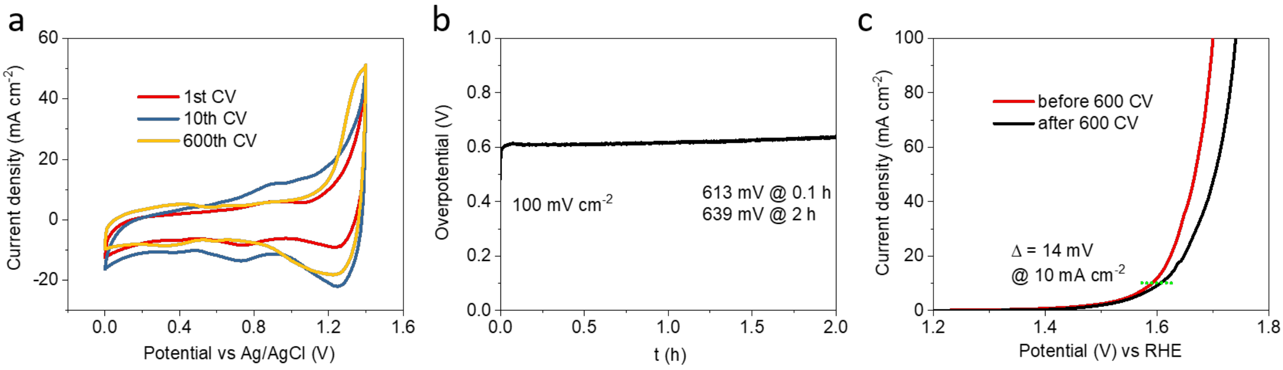

Supplementary Fig. 11. Additional electrochemical data: (a) CV curves of different cycles at 100 mV s^{-1}, (b) stability in chronopotentiometry measurements at 100 mA cm^{-2} and (c) LSV curves before and after 600 CV cycles of 40-Co_3O_4/GPO in 1 M H_2SO_4 electrolyte.

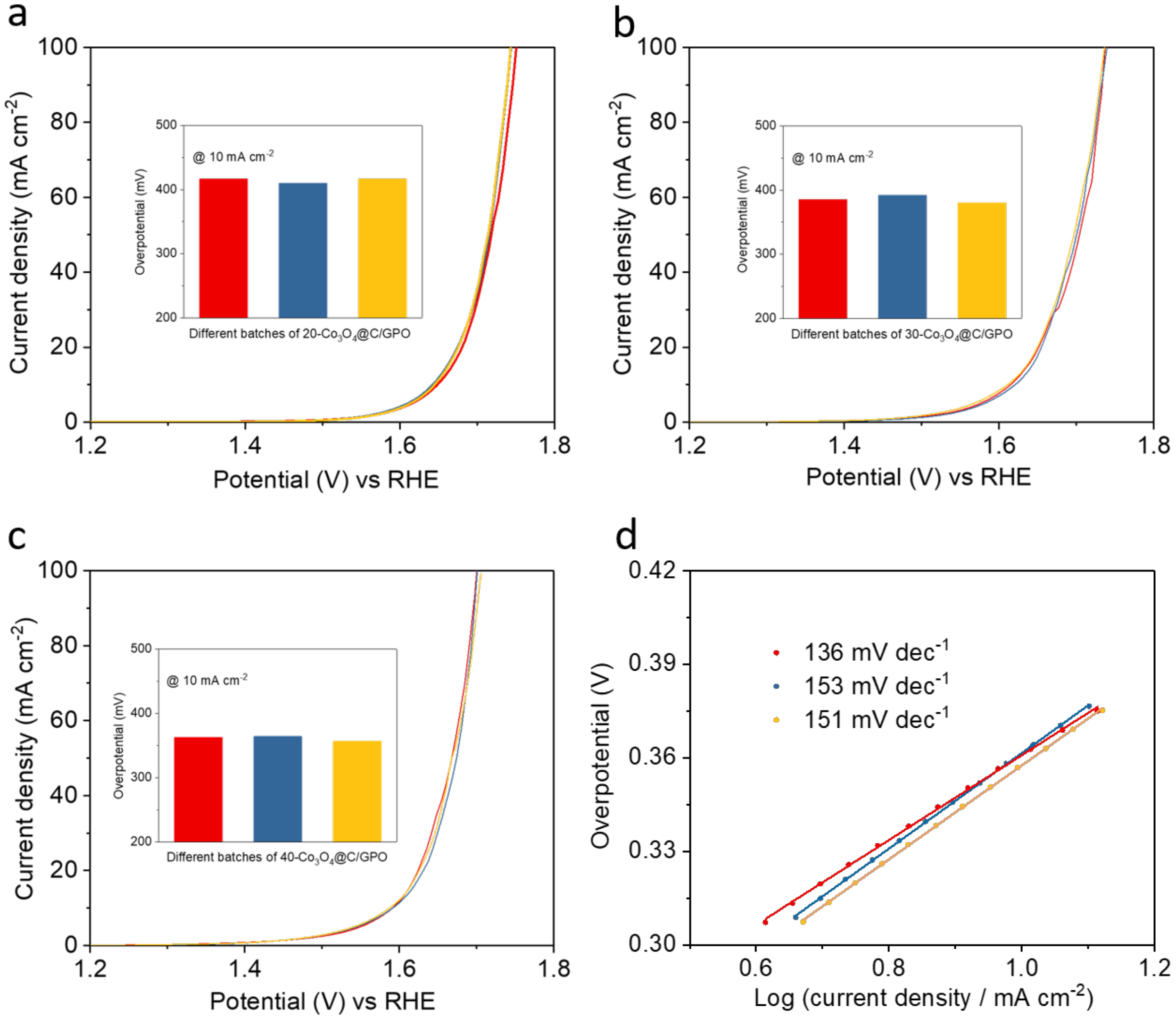

Supplementary Fig. 12. . Additional electrochemical data and analysis: LSV curves of 3 different batches of (a) 20-Co_3O_4/GPO, (b) 30-Co_3O_4/GPO, (c) 40-Co_3O_4/GPO with corresponding overpotentials for 10 mA cm^{-2} in 1 M H_2SO_4 electrolyte inserted, respectively. (d) The Tafel plots of 40-Co_3O_4@C/GPO extracted from LSV data in (c).

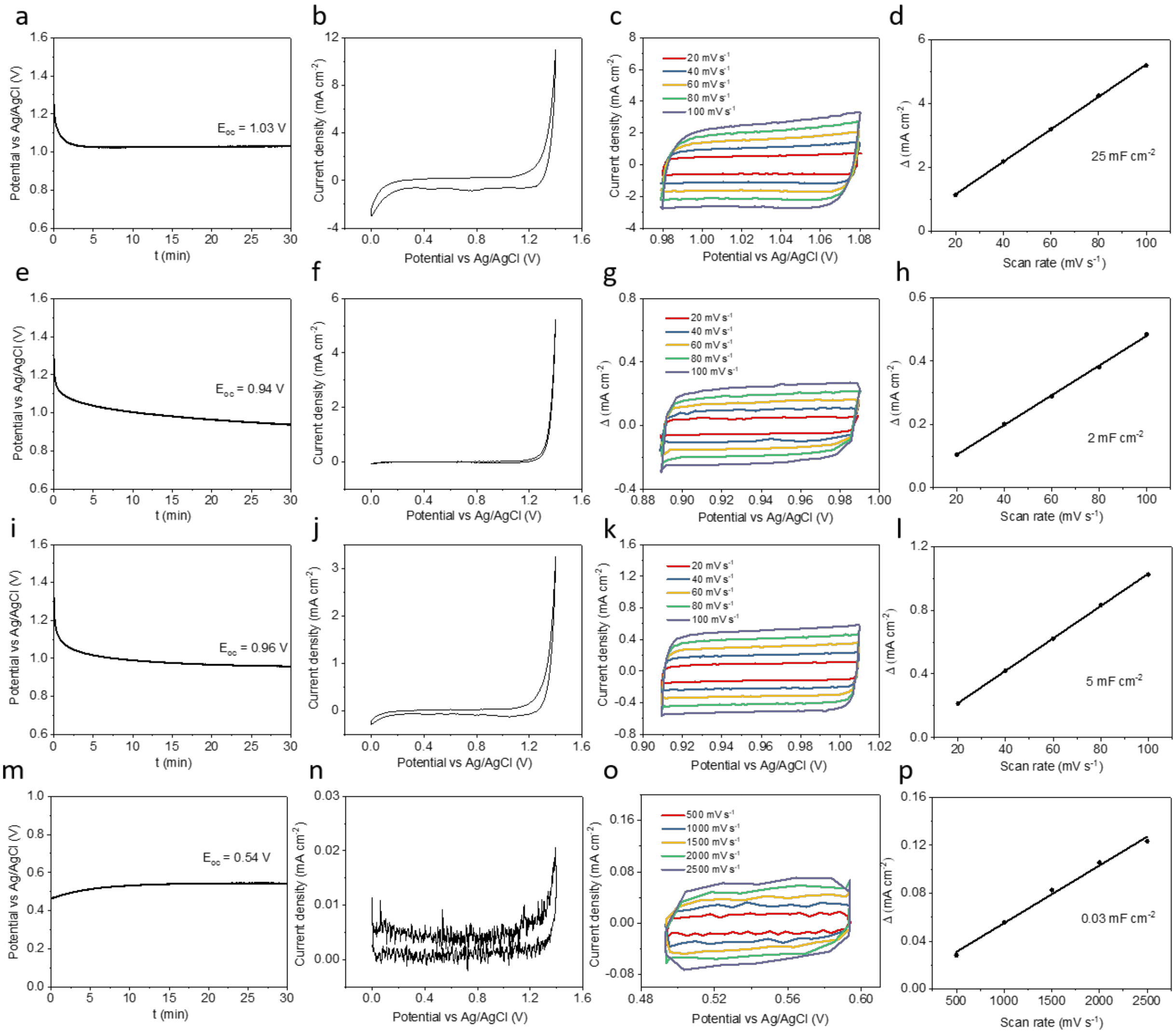

Supplementary Fig. 13. Electrochemical double-layer capacitance (EDLC) measurements: OCP (vs Ag/AgCl) values recording within 30 mins of (a) 20-Co_3O_4@C/GPO, (e) 20-IrO_2/GPO, (i) 20-Co_3O_4/GPO and (m) GPO; CV curves under 10 mV s^{-1} scan rate of (b) 20-Co_3O_4@C/GPO, (f) 20-IrO_2/GPO, (j) 20-Co_3O_4/GPO and (n) GPO; CV curves under different scan rates of (c) 20-Co_3O_4@C/GPO, (g) 20-IrO_2/GPO, (k) 20-Co_3O_4/GPO and (o) GPO; The scan rate dependences of the current density differences Δ of (d) 20-Co_3O_4@C/GPO, (h) 20-IrO_2/GPO, (l) 20-Co_3O_4/GPO and (p) GPO. Note: the current density for GPO electrode under 100 mV s^{-1} was very low and hardly detected so higher scan rates (from 500 to 2500 mV mV s^{-1}) were used to obtain clear value for EDLC determination.

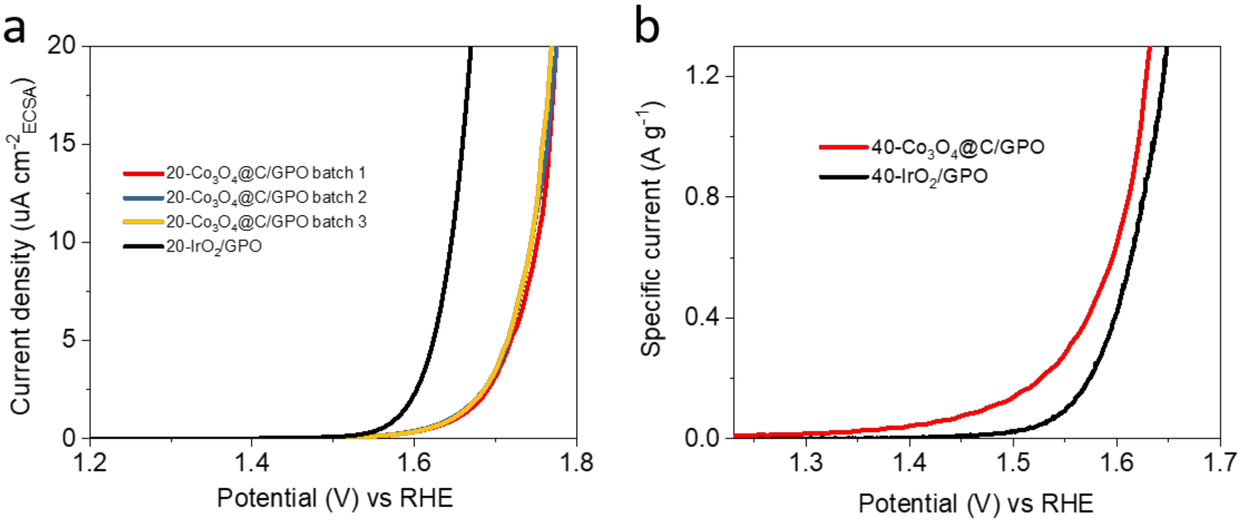

Supplementary Fig. 14. Additional electrochemical data: Comparative potential dependence of the ECSA (a) and mass specific current for IrO_2/GPO and Co_3O_4@C/GPO (b). The electrochemically active surface area (ECSA) was calculated by dividing the electrochemical double-layer capacitance C_{dl} by the specific capacitance C_s of the sample: ECSA = C_{dl} / C_s while a value of 0.035 mF cm^{-2} was suggested for C_s in the reference (*J. Am. Chem. Soc.* **137,** 4347–4357 (2015)).

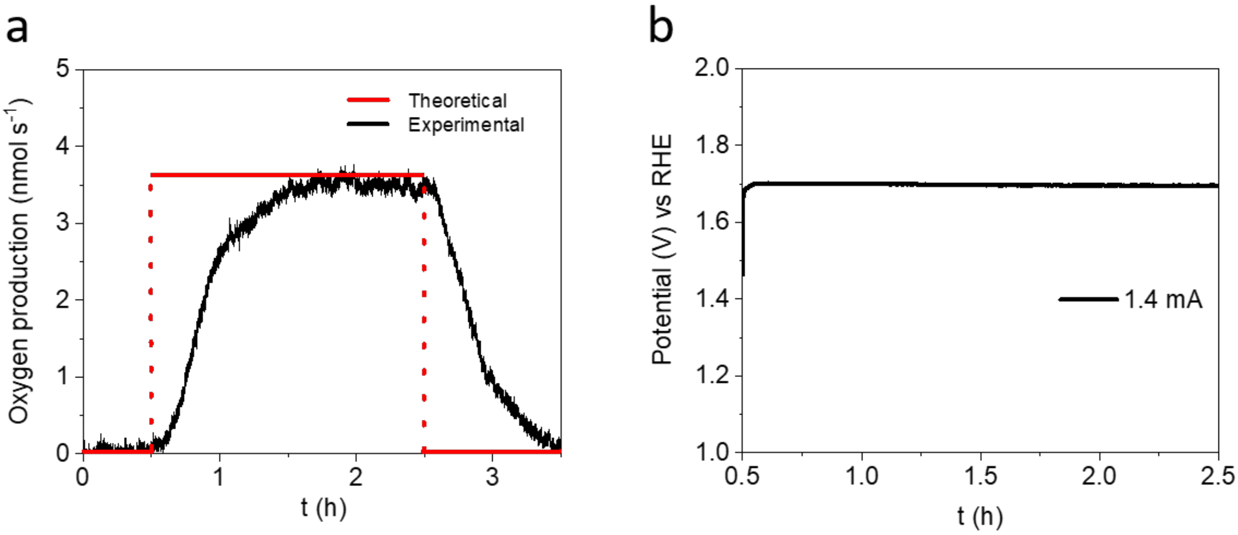

Supplementary Fig. 15. Additional electrochemical data: (a) Time evolution of oxygen production rate in the cell headspace during a chronopotentiometry at 1.4 mA for 2 hours, using 3.3 mL min^{-1} of Ar stream as sweep gas at 20 ºC and 1 atm. Once the chronopotentiometry starts, the O_2 signal rapidly increases, reaching a 3.6 nmol s^{-1} production of O_2 in steady state conditions (reached after 1 hour of operation). This corresponds to a 99% Faradaic efficiency. After the chronopotentiometry, the oxygen signal rapidly decreases, as the chamber is purged with Ar. Integration of all the O_2 detected over time, yields a total of 24.9 µmol of O_2 that corresponds to ≥ 96% of Faradaic efficiency. (b) Chronopotentiometry test for 2 h.

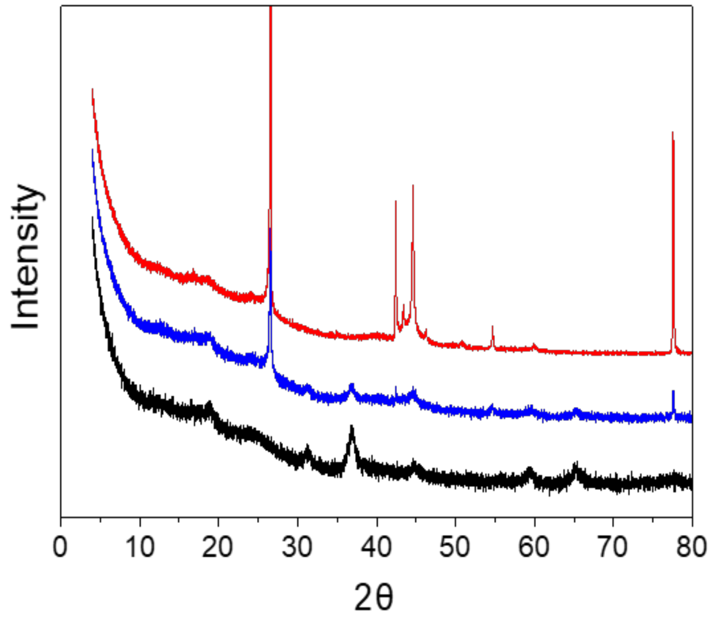

Supplementary Fig. 16. Structural characterization. PXRD patterns of Co_3O_4@C (black), Co_3O_4@C after 2h catalysis, washed with acetone to remove paraffin oil (blue) and commercial graphite (red).

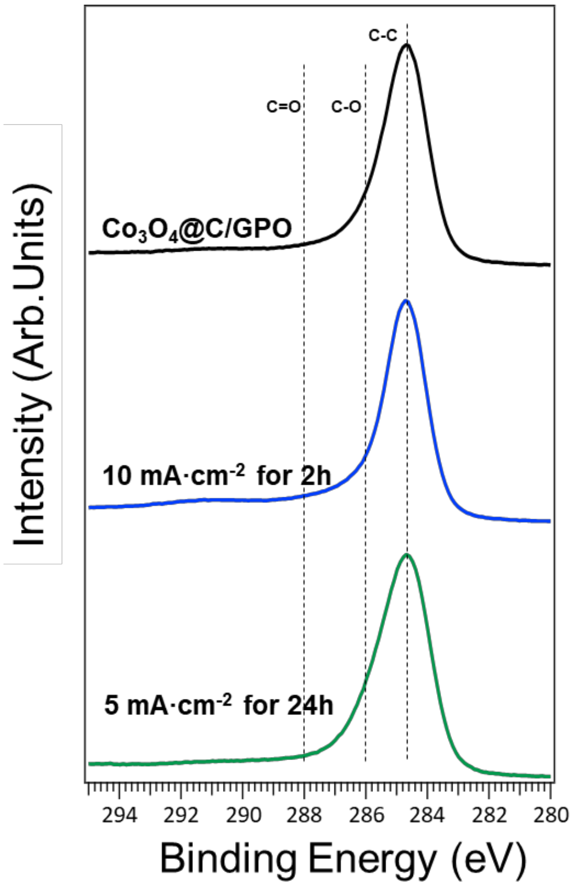

Supplementary Fig. 17. Chemical XPS characterization. C1s XPS spectrum of Co_3O_4@C/GPO (black), Co_3O_4@C/GPO after 2h of electrolysis at 10 mA·cm^{-2} (red) and Co_3O_4@C/GPO after 24h of electrolysis at 5mA·cm^{-2} (green).

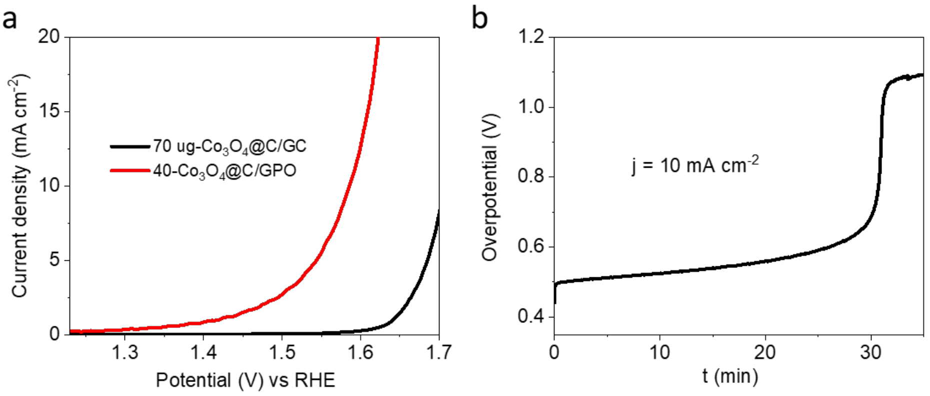

Supplementary Fig. 18. Additional electrochemical data: (a) LSV curves of 40-Co_3O_4@C/GPO and 70 ug-Co_3O_4@C/GC in 1 M H_2SO_4 electrolyte. (b) Chronopotentiometry measurement of 70 ug-Co_3O_4@C/GC at 10 mA cm^{-2}.

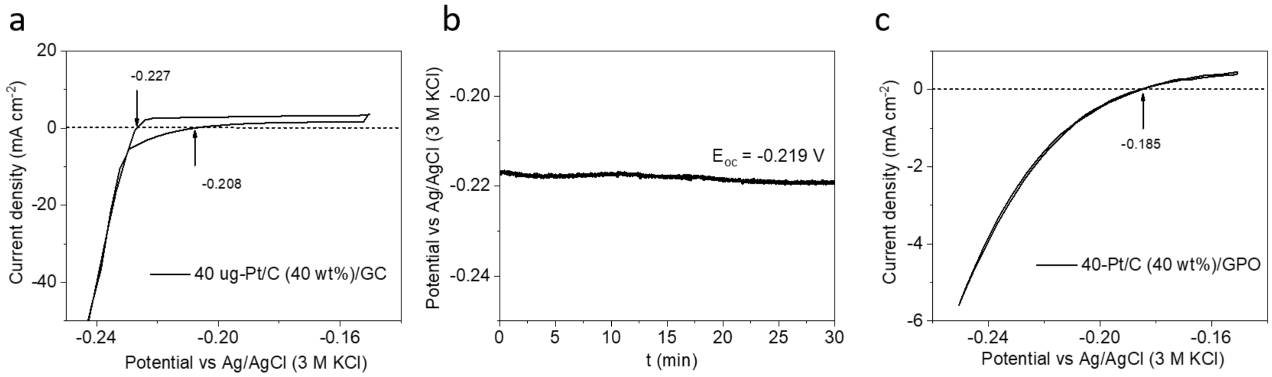

Supplementary Fig. 19. Additional electrochemical data: (a) Calibration of the actual value of the potential of the Ag/AgCl (3 M KCl) vs. the reversible hydrogen electrode (RHE) by using a hydrogen electrode reaction with 40 ug-Pt/C (40 wt%)/GC, the Ag/AgCl electrode as the reference electrode, and carbon rod as the counter electrode in a hydrogen-saturated 1 M H_2SO_4 electrolyte. (b) OCP value recording. (c) CV curve of 40-Pt/C (40 wt%)/GPO with 10 mV s^{-1} scan rate.

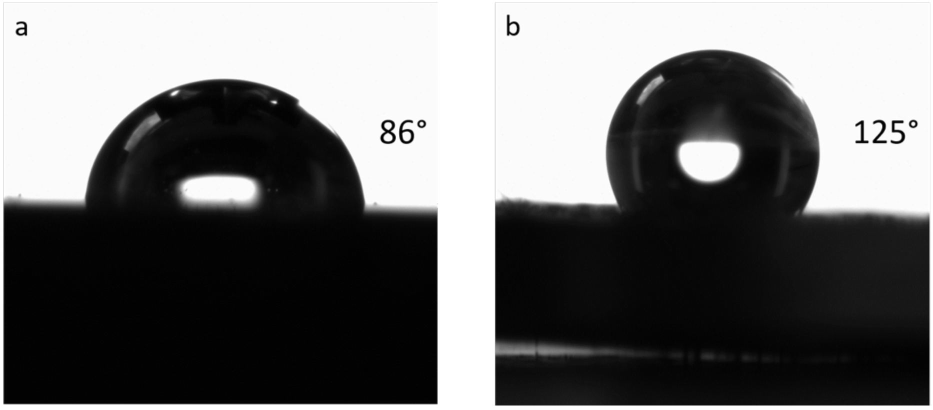

Supplementary Fig. 20. Testing electrode surface hydrophobicity: Contact angle tests of (a) glassy carbon and (b) GPO.

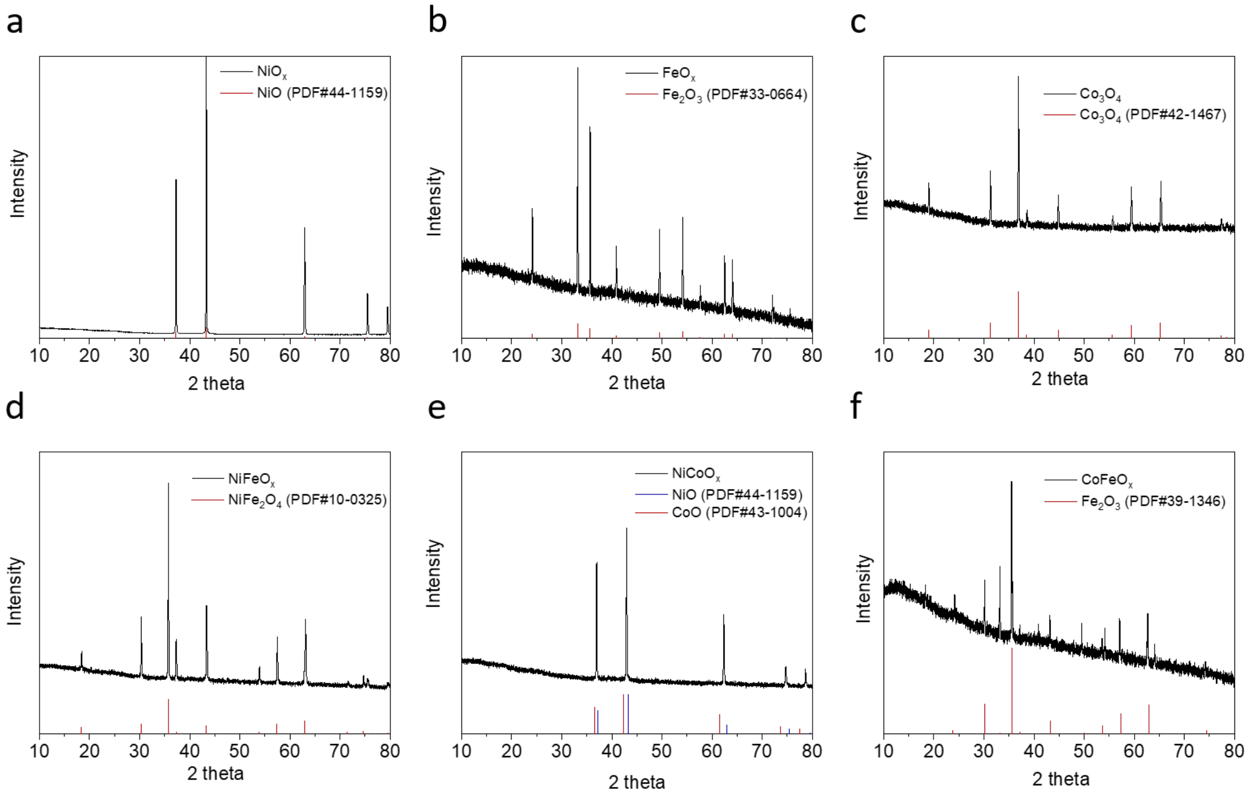

Supplementary Fig. 21. Structural characterization: PXRD patterns of (a) NiO_x, (b) FeO_x, (c) Co_3O_4, (d) $NiFeO_x$, (e) $NiCoO_x$ and (f) $CoFeO_x$.

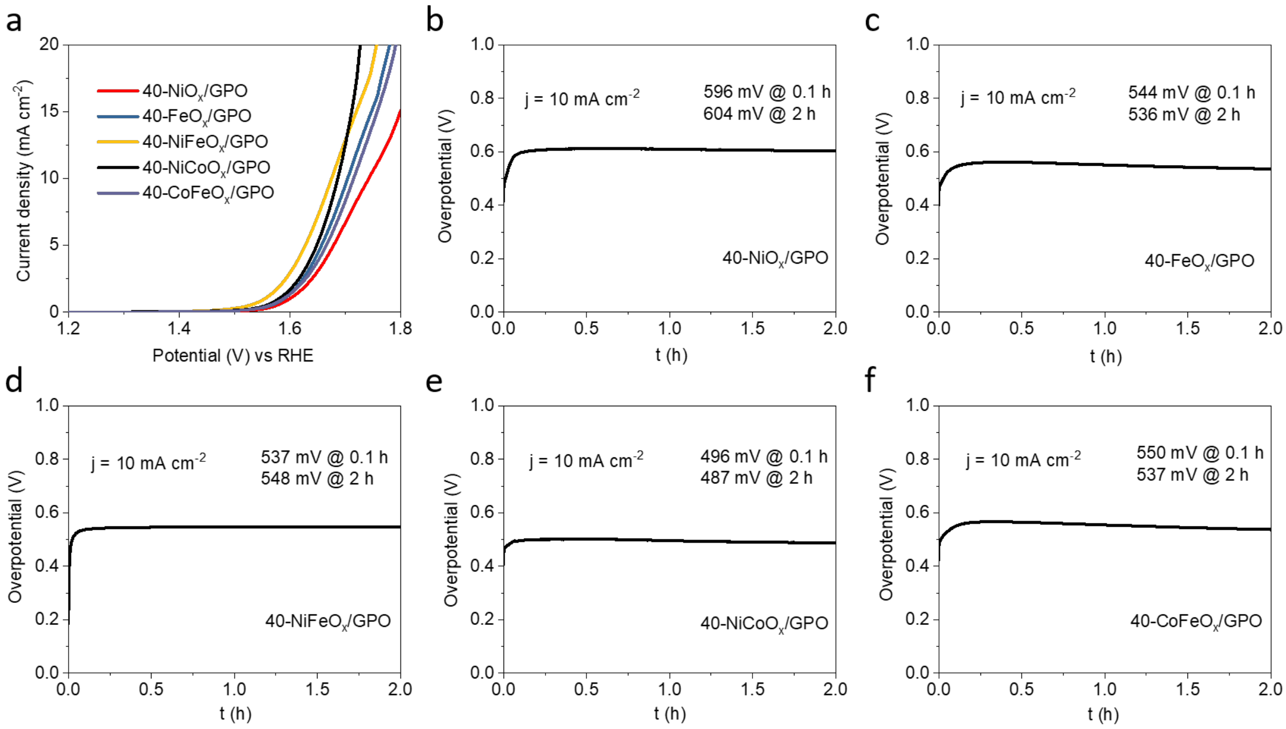

Supplementary Fig. 22. Additional electrochemical data: (a) LSV curves of different oxide electrodes in 1 M H_2SO_4 electrolyte. Chronopotentiometry measurements of (b) 40-NiO_x/GPO, (c) 40-FeO_x/GPO, (d) 40-$NiFeO_x$/GPO, (e) 40-$NiCoO_x$/GPO and (f) 40-$CoFeO_x$/GPO at 10 mA cm^{-2}.

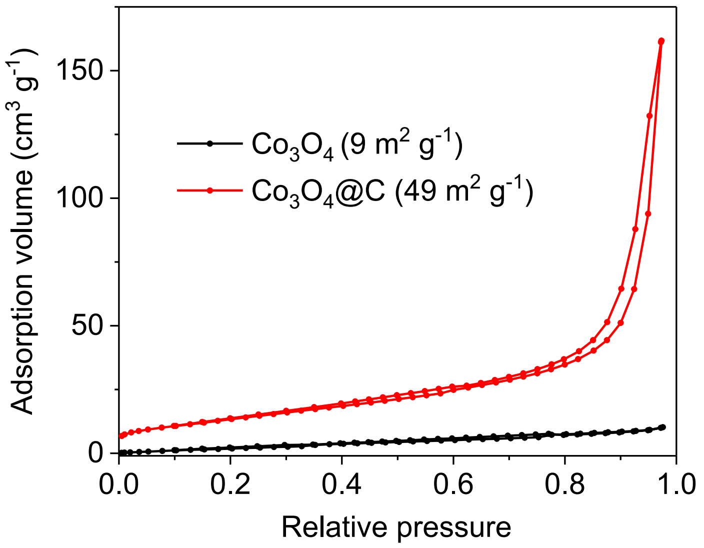

Supplementary Fig. 23. Surface accessibility: Adsorption and desorption isotherms of Co_3O_4@C and Co_3O_4.

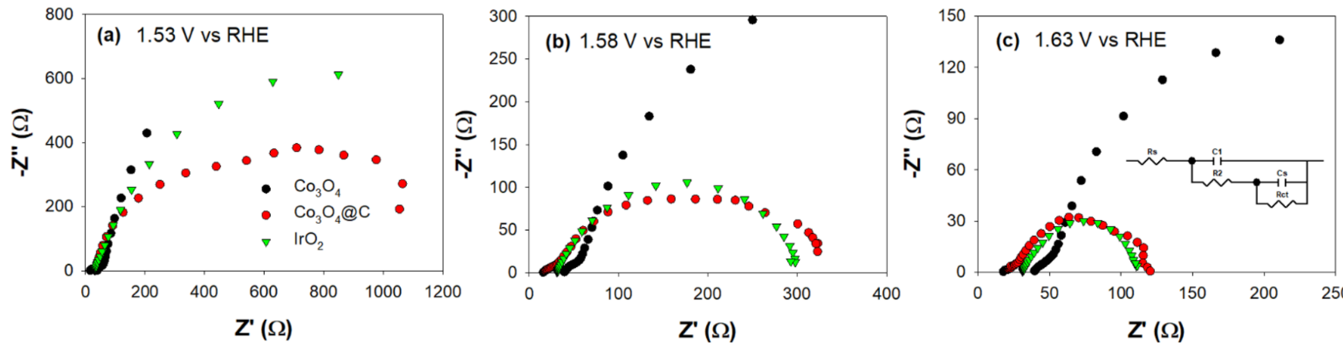

Supplementary Fig. 24. Additional electrochemical data: Nyquist plots from EIS data for Co_3O_4@C/GPO, Co_3O_4/GPO and IrO_2/GPO electrodes at different applied potentials (a) 1.53 V vs RHE, (b) 1.58 V vs RHE and (c) 1.63 V vs RHE. The equivalent circuit model employed to fit the data is also showed as an inset in the right panel (c).

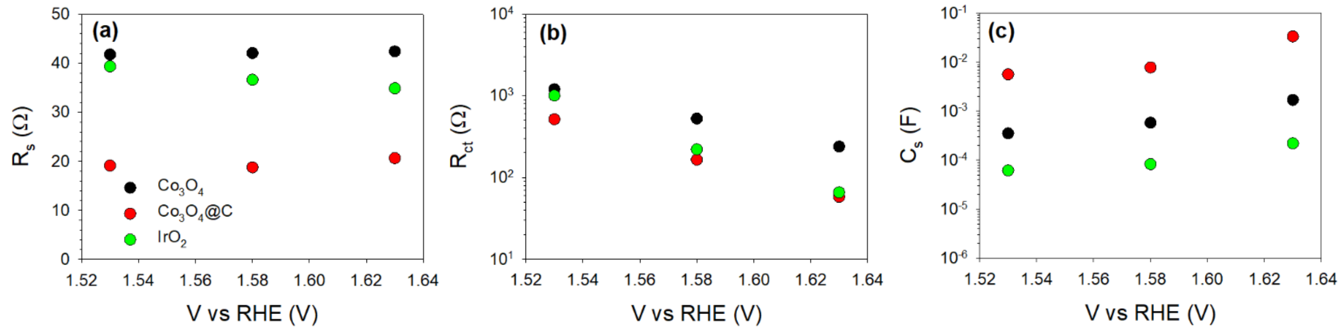

Supplementary Fig. 25. Fitted parameters from the EIS analysis: (a) Series resistance, R_s, (b) Charge transfer resistance, R_{ct} and (c) Surface capacitance, C_S.

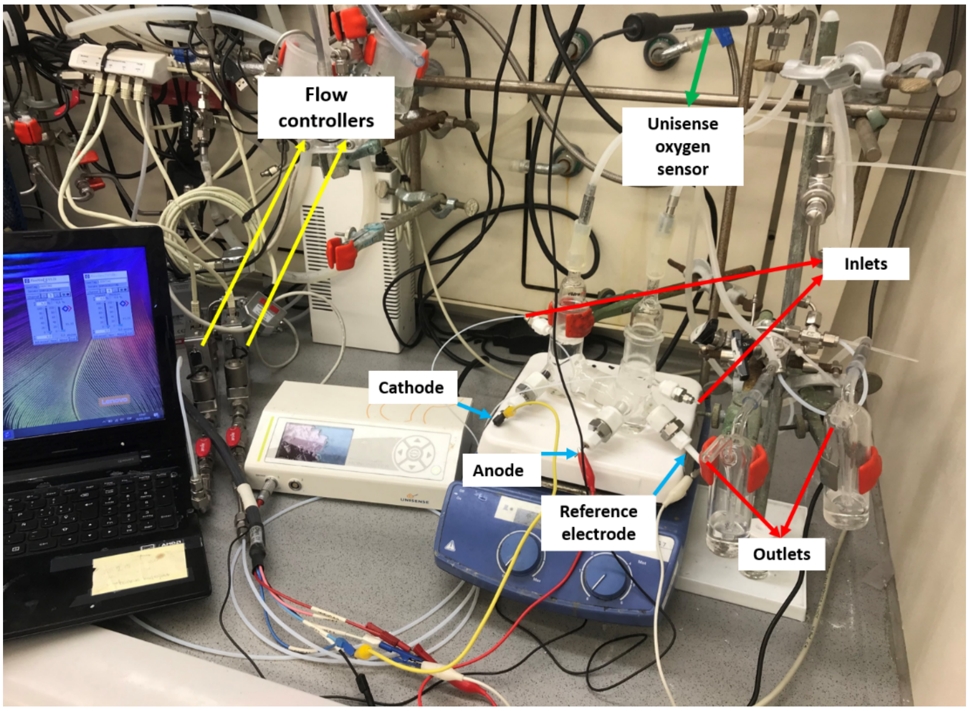

Supplementary Fig. 26. Setup for oxygen evolution measurement. It includes an H-type cell containing the Co_3O_4@C/GPO anode, carbon cathode and Ag/AgCl (3 M KCl) reference electrode connected to the potentiostat, the Unisense oxygen sensor connected to the anodic side headspace and the gas flow controlling system employed to feed Ar carrier gas to the cell.

Supplementary Table 1. The actual ratios of carbon, nitrogen, hydrogen and cobalt determined by elemental analysis.

	Carbon (wt%)	Nitrogen (wt%)	Hydrogen (wt%)	Cobalt (wt%)
Co_3O_4@C	8.28	0.26	0.47	62

Supplementary Table 2. OER activity for working electrodes from LSV curves in 1 M H_2SO_4 (pH 0.3).

	η (mV) @ 1 mA cm^{-2}	η (mV) @ 5 mA cm^{-2}	η (mV) @ 10 mA cm^{-2}	η (mV) @ 20 mA cm^{-2}
40-Co_3O_4@C/GPO	190	313	356	393
40-IrO_2/GPO	291	344	368	396

Supplementary Table 3. Elemental analysis (ICP-MS) of cobalt before and after 2 h electrocatalytic water oxidation at a constant current density of 10 mA cm^{-2} in 1 M H_2SO_4 of 40 mL.

	Cobalt amount (μg/L)
1 M H_2SO_4 before	–
1 M H_2SO_4 after	756

Supplementary Table 4. Performance parameters for different electrocatalysts for OER in strong acidic electrolytes.

Catalyst	[H_2SO_4]	Scan rate (mV s^{-1})	η (mV)[a]	Tafel slope (mV dec^{-1})	Stability	loading (mg cm^{-2})	*S*	ref.
Co_3O_4@C/GPO	1 M	1	356	139	43 h[a]	20	25	this work
[Co-POM]/CP	1 M	1	361	97	24 h @ η = 250 mV	20	~	2
IrO_2	1 M	1	458	66	24 h @ η = 250 mV	26	~	2
ATO/CoHFe	0.1 M	50	770	~	~	0.61	~	3
$IrO_2/SrIrO_3$	0.5 M	10	280	~	30 h[a]	~	~	4
$CoFePbO_x$	1 M	~	620	~	12 h @ 2.03 V	~	~	5
MnO_2	0.1 M	1	428	80	8000 h[a]	36	~855	6
$Ni_{0.5}Mn_{0.5}Sb_{1.7}O_x$	1 M	10	672	60	168 h[a]	~0.18	~6194	7
$Co_{0.05}Fe_{0.95}O_y$	0.5 M	10	650	110	50 h[a]	1	~	8
$Mn_xSb_{1-x}O_z$	1 M	20	508	75	2 h[a]	~	71	9
Co_3O_4/FTO	0.5 M	2	490	80	12 h[a]	~	~847	10
$W_{0.57}Ir_{0.43}O_{3-\delta}$	1 M	20	370	125	2000 s[a]	~	~	11
Ti-MnO_2	0.05 M	5	~540[b]	170	2 h @ 1.9 V	~	~16230	12
$Ni_{40}Fe_{40}P_{20}$	0.05 M	5	540	-	30 h[a]	~	~	13
c-Fe_2O_3	0.5 M	10	650	56	24 h[a]	1	~174	14
P-NSC/ $Ni_4Fe_5S_8$-1000	0.5 M	2	~560	72	10000 cycles[c]	0.4	~	15
$Y_{1.85}Zn_{0.15}Ru_2O_{7-\delta}$	0.5 M	10	291	37	2000 cycles @ 1.55 V	3	~	16
1T-MoS_2	0.5 M	5	420	322	2 h[a]	1	~	17
Ir-ND/ATO	0.05 M	5	~400	56	15 h[a]	0.01	~	18
Ir/TiO_x	0.5 M	6	~320	53	~	~	~	19
IrO_x/ATO	0.05 M	5	~420	60	15 h[c]	0.01	12307	20

[a] @10 mA cm^{-2}
[b] @2 mA cm^{-2}
[c] @ 1 mA cm^{-2}

Supplementary Table 5. Mass loading of catalysts in working electrodes.

Catalysts	Co_3O_4@C	IrO_2	Co_3O_4	NiO_x	FeO_x	$NiFeO_x$	$CoFeO_x$	$NiCoO_x$
Mass loading (mg cm^{-2})	20	25	19	24	20	22	23	22

Supplementary Table 6. Ohmic drop values determined by the automatic current interrupt (CI) software and actual mass of catalysts in working electrodes.

	40-Co_3O_4@C/GPO	30-Co_3O_4@C/GPO	20-Co_3O_4@C/GPO	40-IrO_2/GPO	30-IrO_2/GPO	20-IrO_2/GPO
Ohmic drop (Ω)	19	37	27	25	28	25
Total electrode mass (mg)*	39.9	39.1	40.7	47.7	45.2	43.8
Total mass of Co_3O_4@C in the electrode (mg)*	11	9	7	14	10	7

*total mass of the composite: Co_3O_4@C + graphite + paraffin oil; 1/8 of total mass is used for activity comparison as suggested by Ref.2.

Supplementary References

66. K. S. Park, Z. Ni, A. P. Côté, J. Y. Choi, R. Huang, F. J. Uribe-Romo, H. K. Chae, M. O'Keeffe and O. M. Yaghi, *Proc. Natl. Acad. Sci. U. S. A*. 2006, **103**, 10186–10191.
67. M. Blasco-Ahicart, J. Soriano-Lopez, J. J. Carbo, J. M. Poblet and J. R. Galan-Mascaros, *Nat. Chem*., 2018, **10**, 24–30.
68. B. Rodríguez-García, Á. Reyes-Carmona, I. Jiménez-Morales, M. Blasco-Ahicart, S. Cavaliere, M. Dupont, D. Jones, J. Rozière, J. R. Galán-Mascarós and F. Jaouen, *Sustain. Energy Fuels*, 2018, **2**, 589–597.
69. L. C. Seitz, C. F. Dickens, K. Nishio, Y. Hikita, J. Montoya, A. Doyle, C. Kirk, A. Vojvodic, H. Y. Hwang, J. K. Norskov and T. F. Jaramillo, *Science*, 2016, **353**, 1011–1014.
70. M. Chatti, J. L. Gardiner, M. Fournier, B. Johannessen, T. Williams, T. R. Gengenbach, N. Pai, C. Nguyen, D. R. MacFarlane, R. K. Hocking and A. N. Simonov, *Nat. Catal*., 2019, **2**, 457–465.
71. A. Li, H. Ooka, N. Bonnet, T. Hayashi, Y. Sun, Q. Jiang, C. Li, H. Han and R. Nakamura, *Angew. Chemie - Int. Ed*., 2019, **58**, 5054–5058.
72. I. A. Moreno-Hernandez, C. A. Macfarland, C. G. Read, K. M. Papadantonakis, B. S. Brunschwig and N. S. Lewis, *Energy Environ. Sci.,* 2017, **10**, 2103–2108.
73. W. L. Kwong, C. C. Lee, A. Shchukarev and J. Messinger, *Chem. Commun*., 2019, **55**, 5017–5020.
74. L. Zhou, A. Shinde, J. H. Montoya, A. Singh, S. Gul, J. Yano, Y. Ye, E. J. Crumlin, M. H. Richter, J. K. Cooper, H. S. Stein, J. A. Haber, K. A. Persson and J. M. Gregoire, *ACS Catal*., 2018, **8**, 10938–10948.
75. J. S. Mondschein, J. F. Callejas, C. G. Read, J. Y. C. Chen, C. F. Holder, C. K. Badding and R. E. Schaak, *Chem. Mater.,* 2017, **29**, 950–957.
76. S. Kumari, B. P. Ajayi, B. Kumar, J. B. Jasinski, M. K. Sunkara and J. M. Spurgeon, *Energy Environ. Sci.*, 2017, **10**, 2432–2440.
77. R. Frydendal, E. A. Paoli, I. Chorkendorff, J. Rossmeisl and I. E. L. Stephens, *Adv. Energy Mater*., 2015, **5**, 1500991.
78. F. Hu, S. Zhu, S. Chen, Y. Li, L. Ma, T. Wu, Y. Zhang, C. Wang, C. Liu, X. Yang, L. Song, X. Yang and Y. Xiong, *Adv. Mater*., 2019, **29**, 1606570.
79. W. L. Kwong, C. C. Lee, A. Shchukarev, E. Bjorn, J. Messinger, *J. Catal*., 2018, **365**, 29-35.
80. Q. Hu, G. Li, X. Liu, B. Zhu, G. Li, L. Fan, X. Chai, Q. Zhang, J. Liu, C. He, J. Mater. Chem. A, 2019, **7**, 461–468.
81. Q. Feng, Q. Wang, Z. Zhang, Y. Xiong, H. Li, Y. Yao, X.-Z. Yuan, M. C. Willams, M. Gu, H. Chen, H. Li, H. Wang, *Appl. Catal. B*, 2019, **224**, 494-501.
82. J. Wu, M. Liu, K. Chatterjee, K. P. Hackenberg, J. Shen, X. Zou, Y. Yan, J. Gu, Y. Yang, J. Lou, P. M. Ajayan, *Adv. Mater. Interfaces*, 2016, **3**, 1500669.
83. H.-S Oh, H. N. Nong, T. Reier, M. Gliech, P. Strasser, *Chem. Sci*., 2015, **6**, 3321-3328.
84. M. Bernicke, D. Bernsmeier, B. Paul, R. Schmack, A. Bergmann, P. Strasser, E. Ortel, P. Kraehnert, *J. Catal*., 2019, **376**, 209-218.
85. H.-S Oh, H. N. Nong, T. Reier, A. Bergmann, M. Gliech, J. F. Araujo, E. Willinger, R. Schlogl, D. Teschner, P. Strasser, *J. Am. Chem. Soc.,* 2016, **138**, 12552-12563.
