# Supplementary figures and images for: Sustainable oxygen evolution electrocatalysis in aqueous 1 M H2SO4 with earth abundant nanostructured Co3O4
Source: Nat Commun. 2022 Jul 27;13:4341. doi: 10.1038/s41467-022-32024-6 (PMC9329283; doi:10.1038/s41467-022-32024-6)

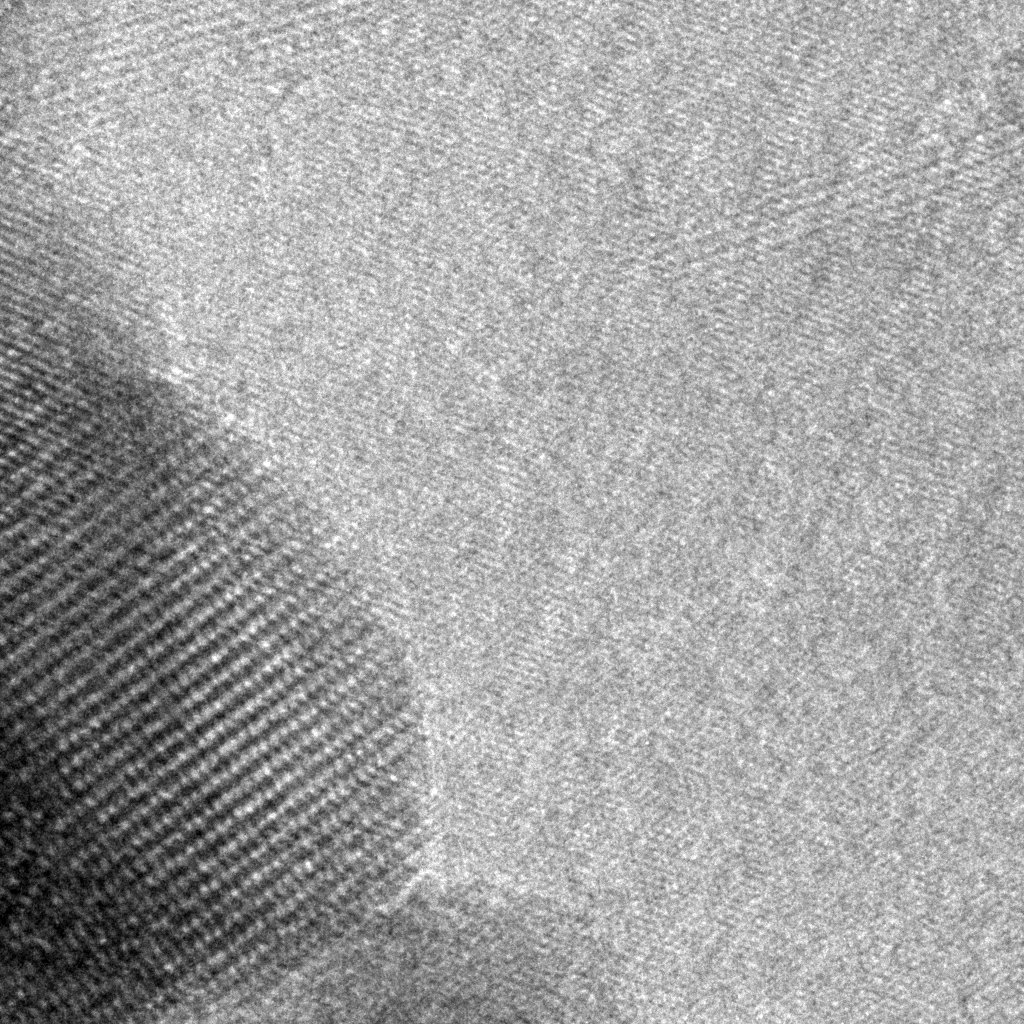

Supplement: Supplementary file 3 — Source Data [file 41467_2022_32024_MOESM3_ESM.zip › Source Data/SourceData1/Fig6.jpg]

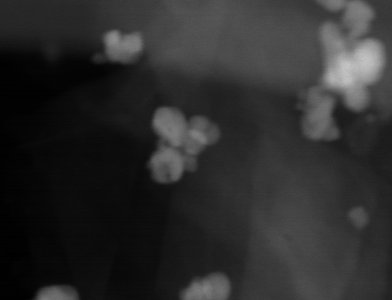

Supplement: Supplementary file 3 — Source Data [file 41467_2022_32024_MOESM3_ESM.zip › Source Data/SourceData1/FigS8_STEM.jpg]

## Slide 1
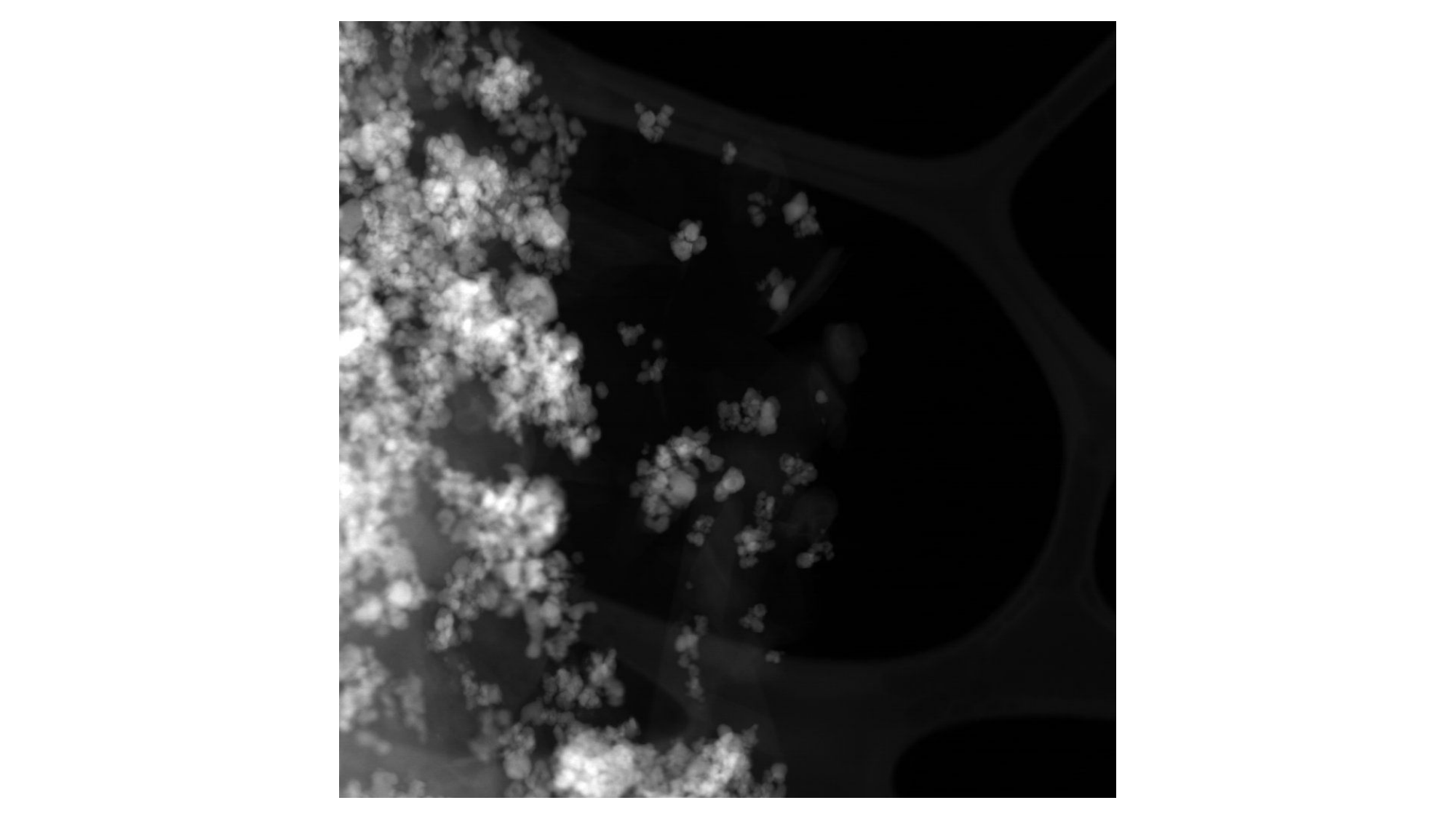

## Slide 2
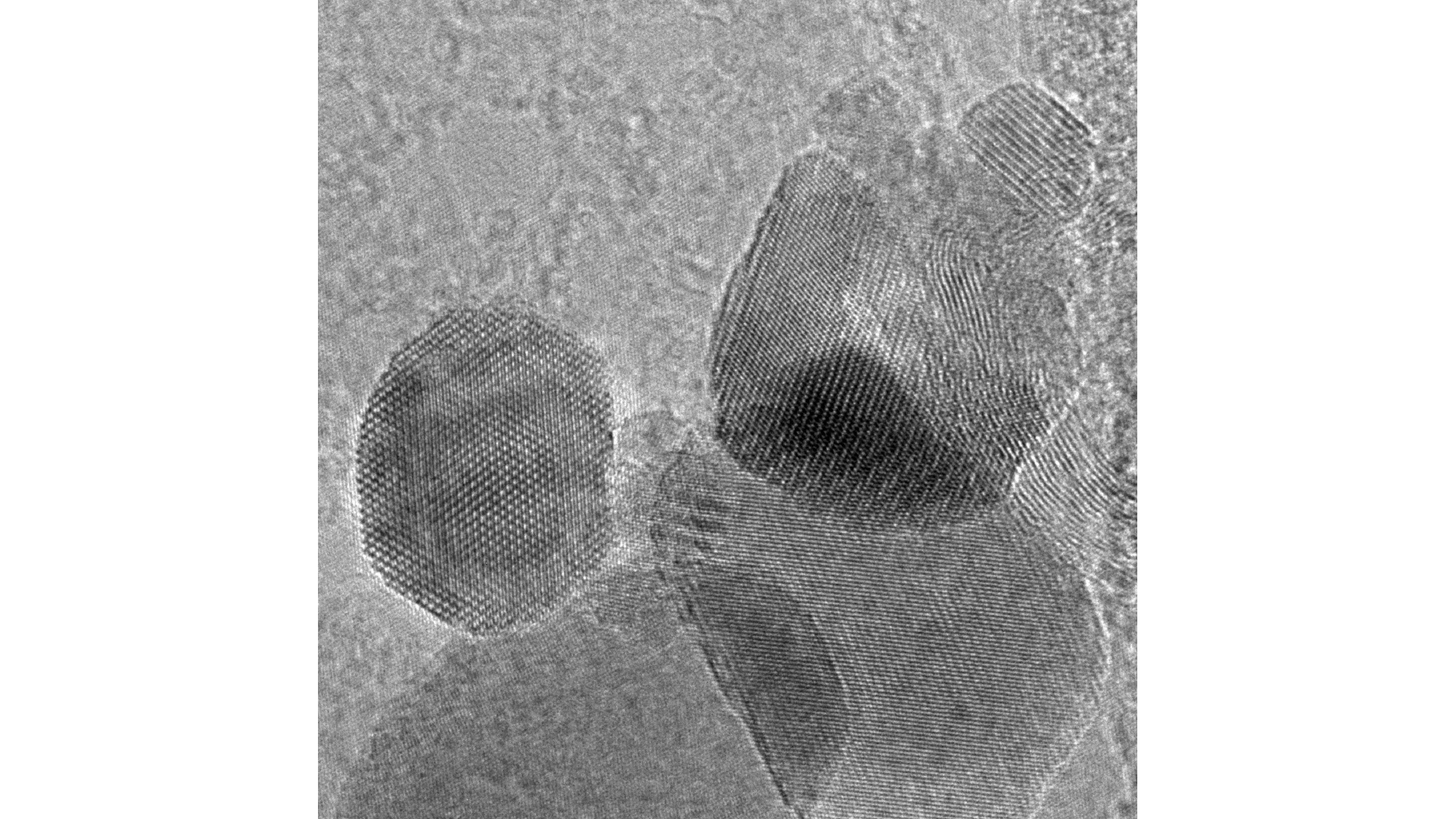

## Slide 3
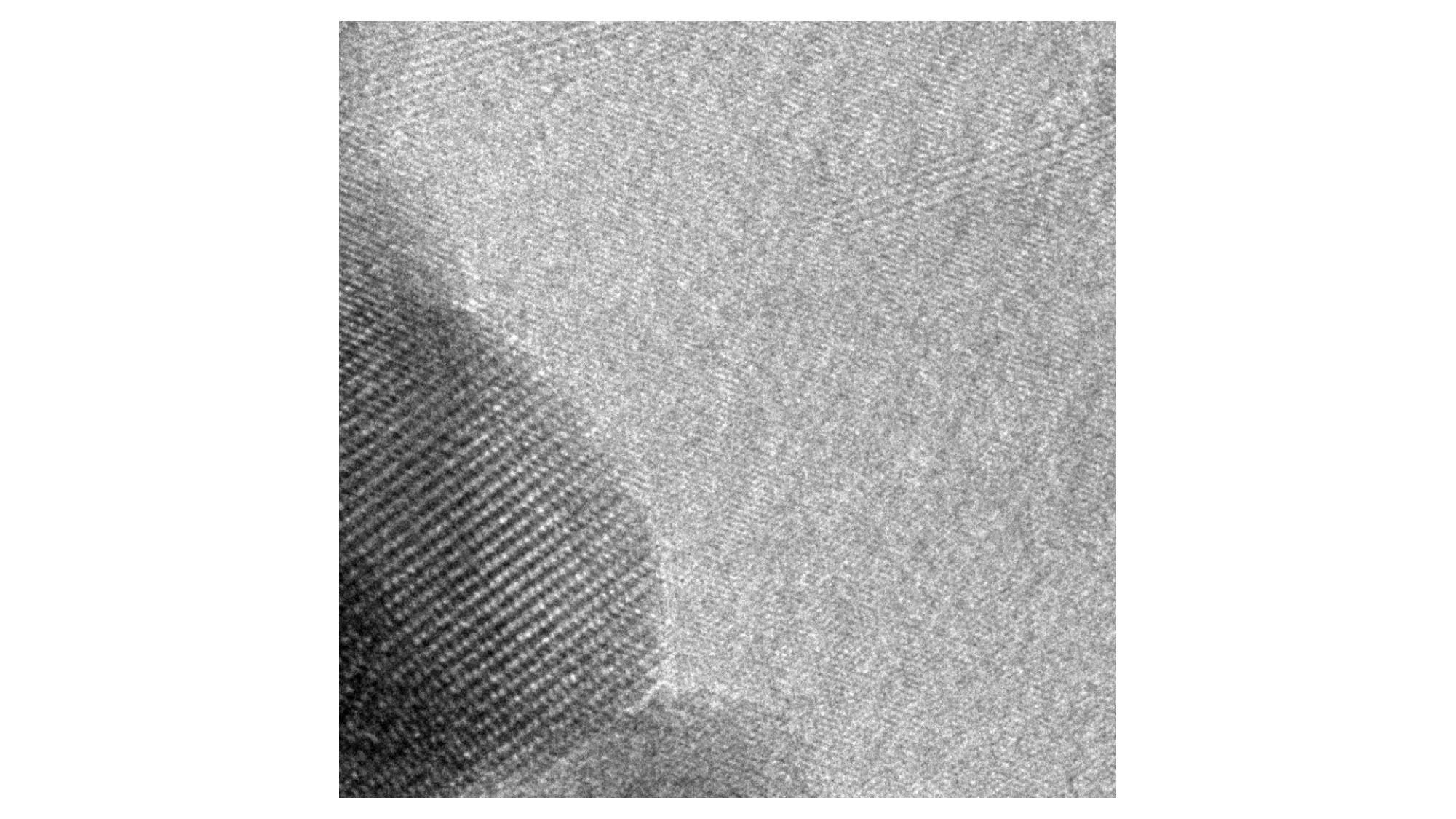

## Slide 4
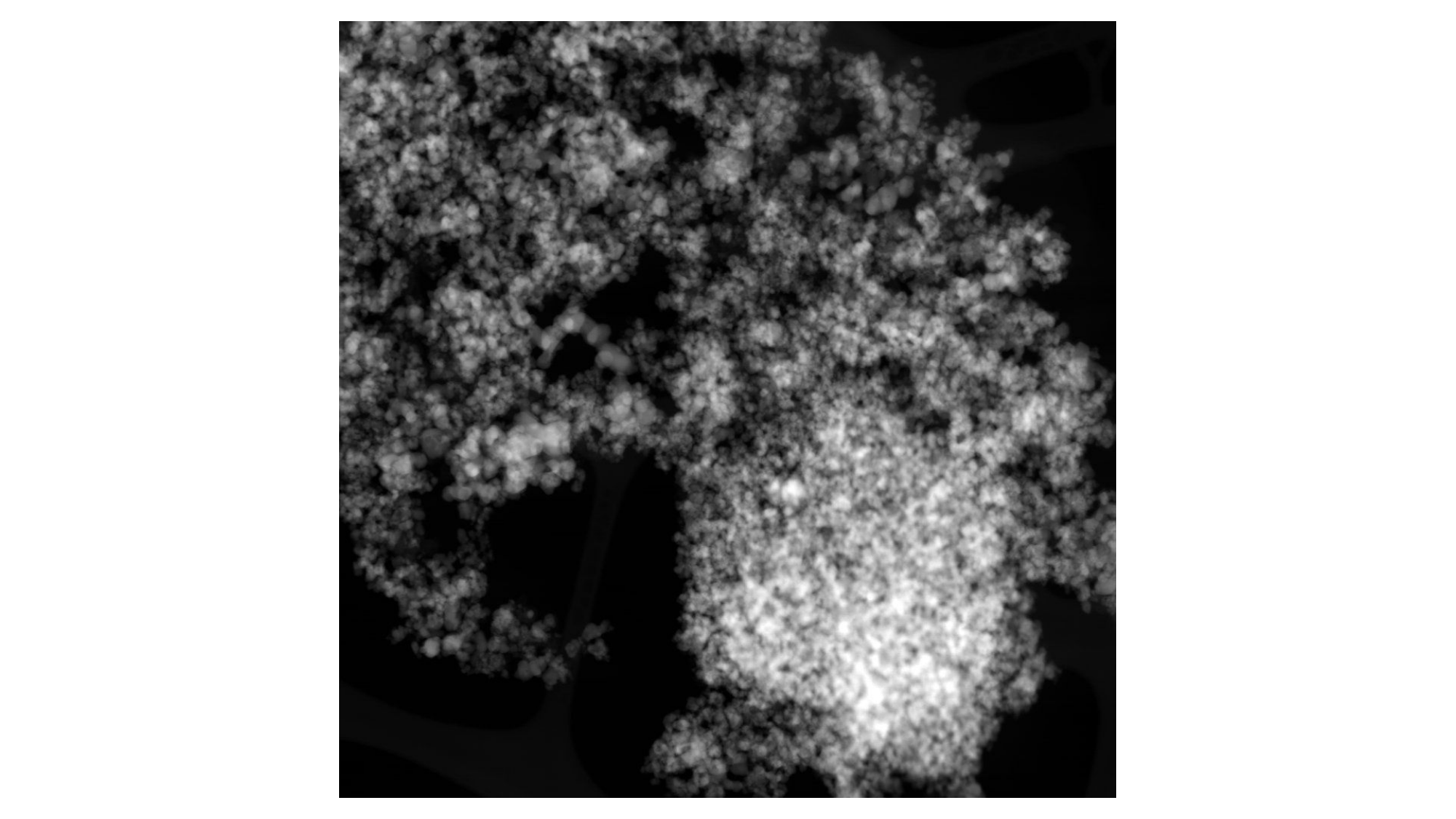

## Slide 5
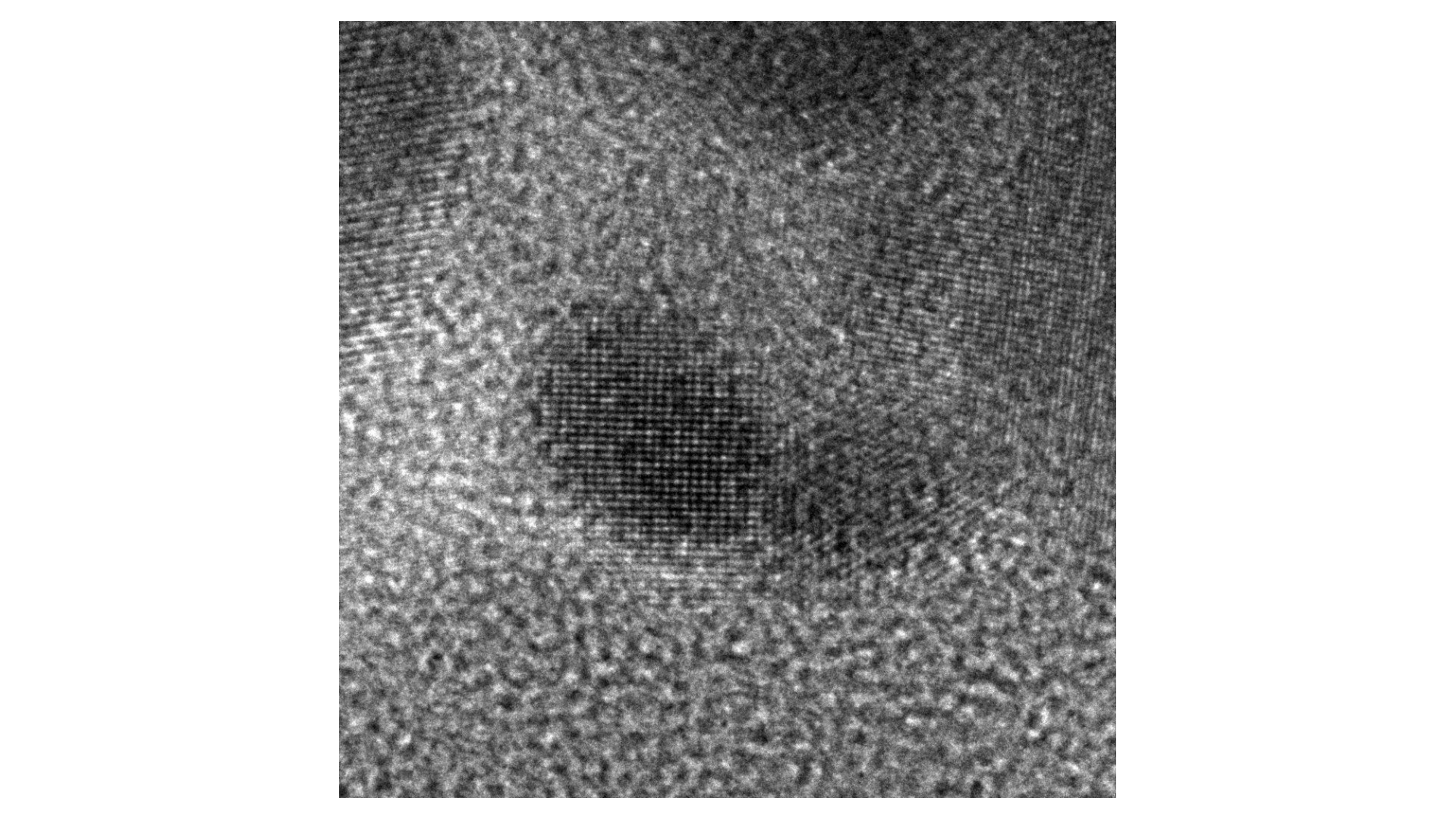

## Slide 6
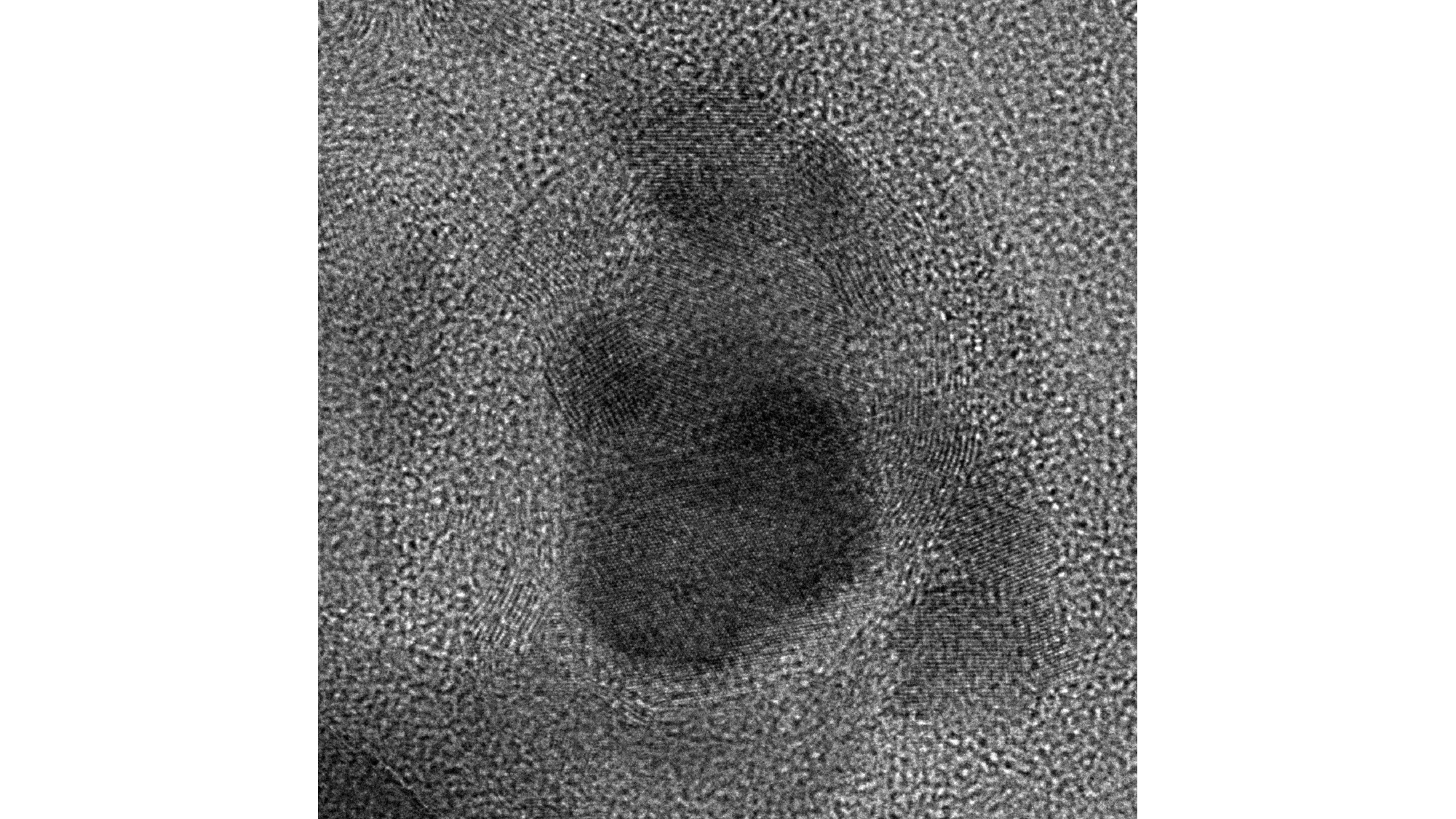

## Slide 7
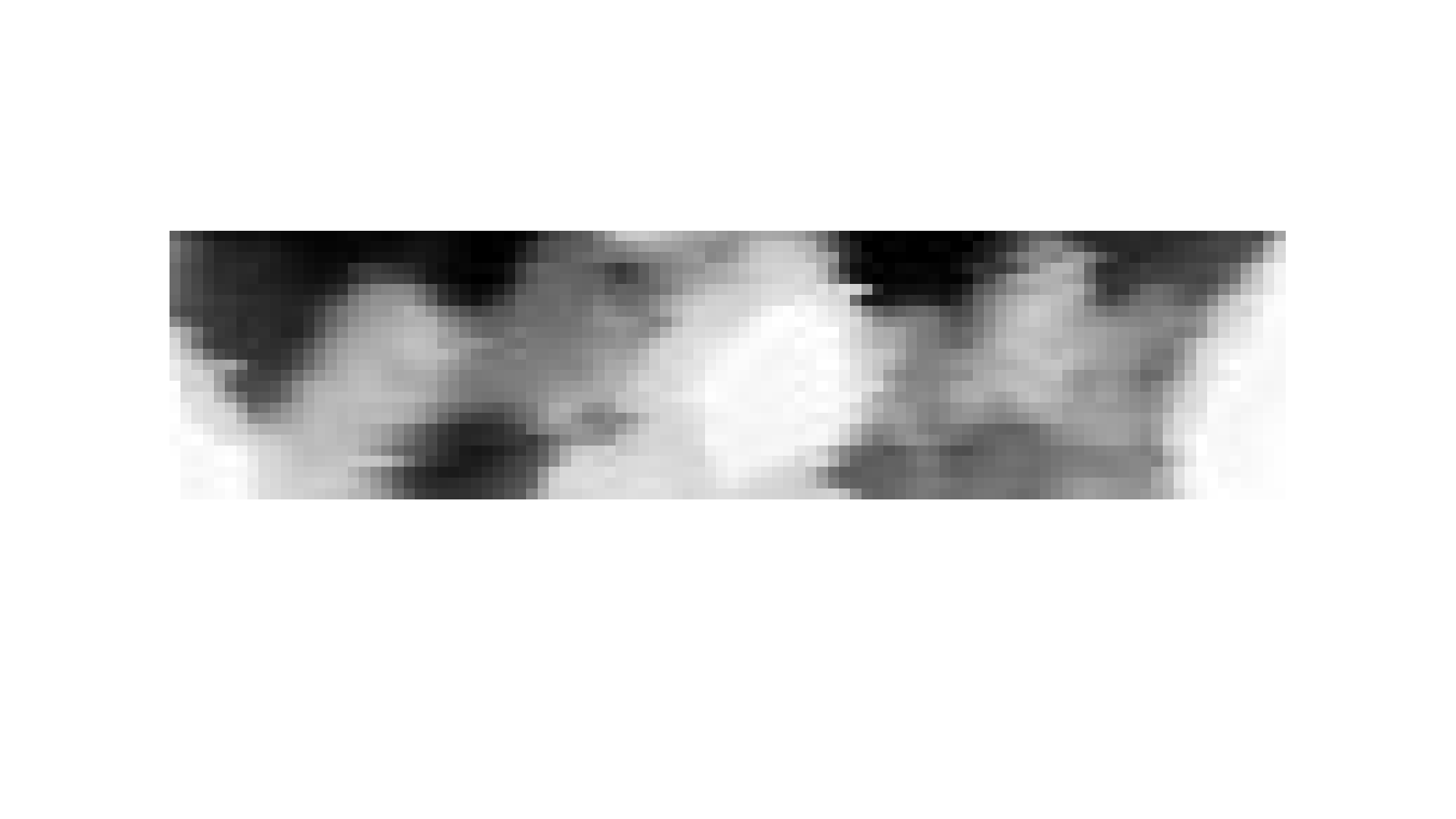

## Slide 8
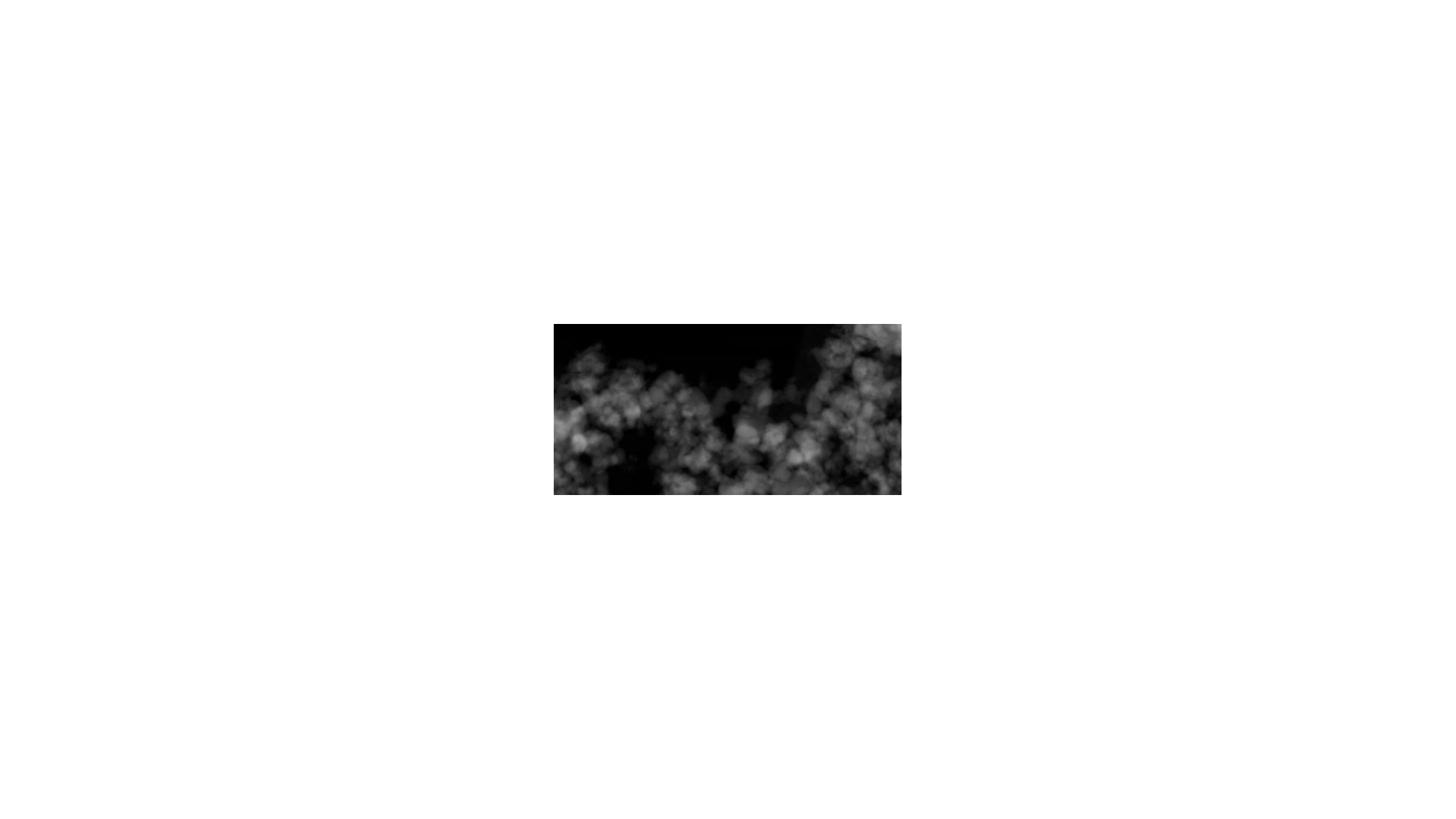

## Slide 9
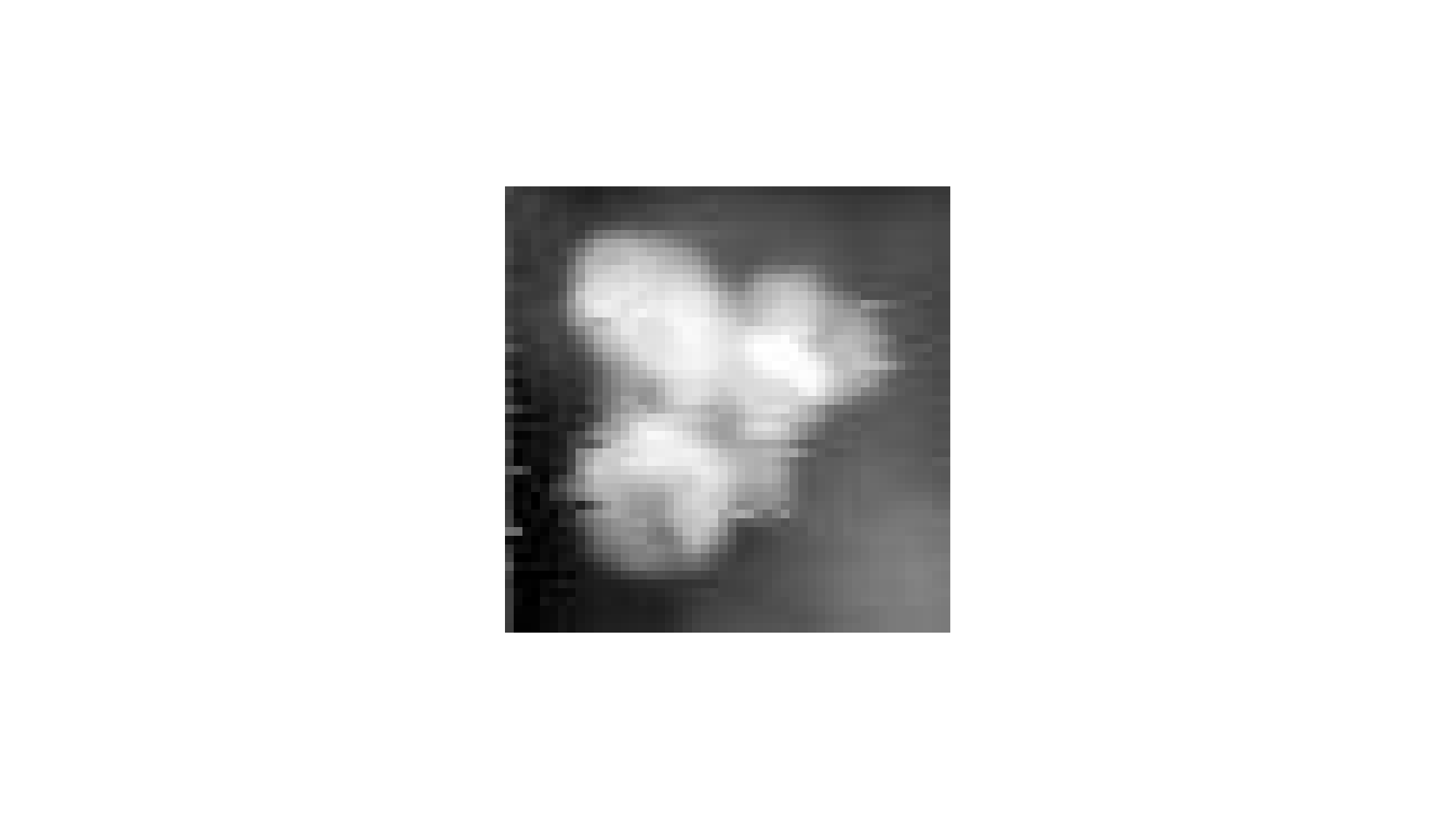

## Slide 10
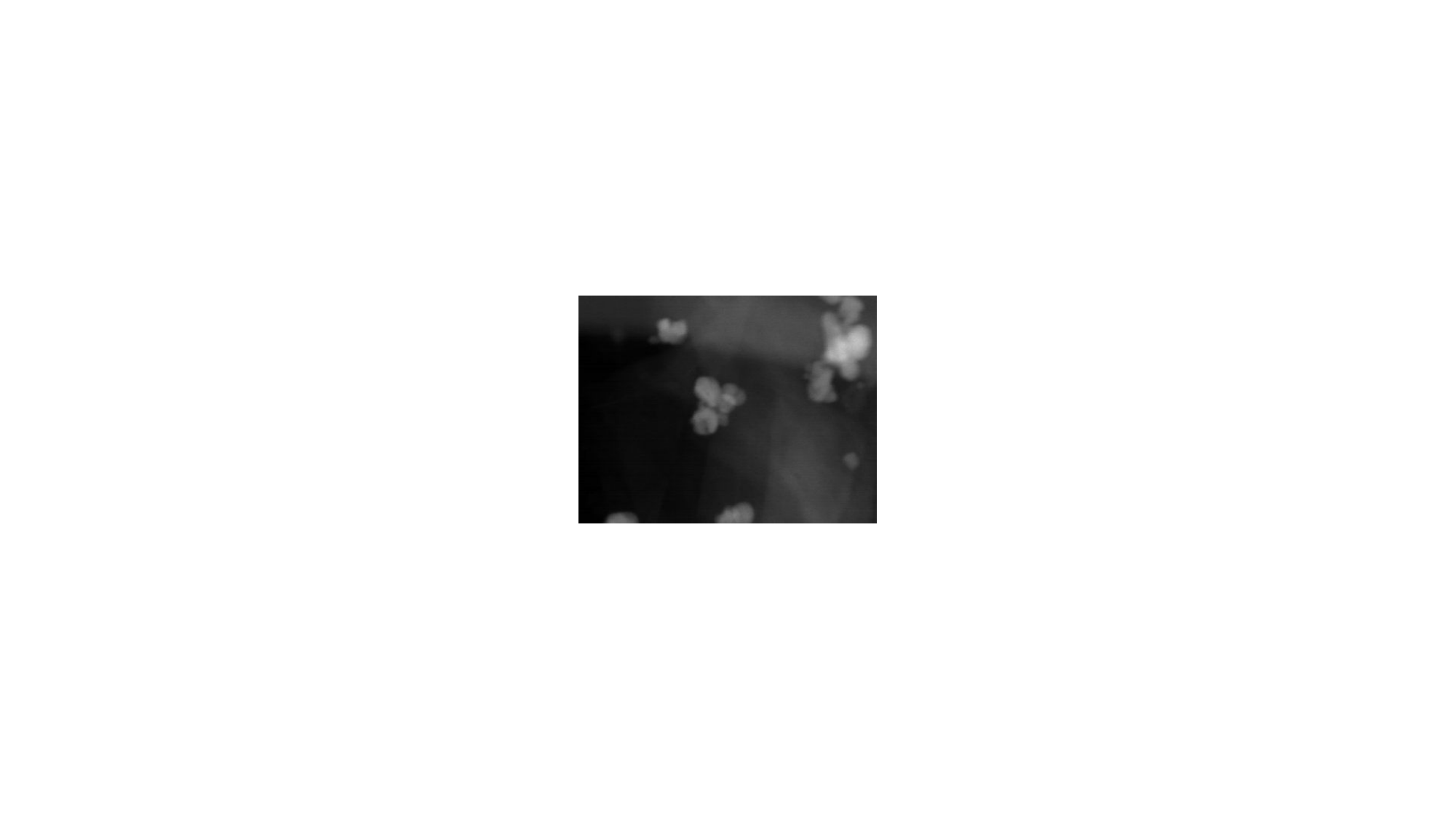

Supplement: Supplementary file 3 — Source Data [file 41467_2022_32024_MOESM3_ESM.zip › Source Data/SourceData1/DurceData1.pptx]

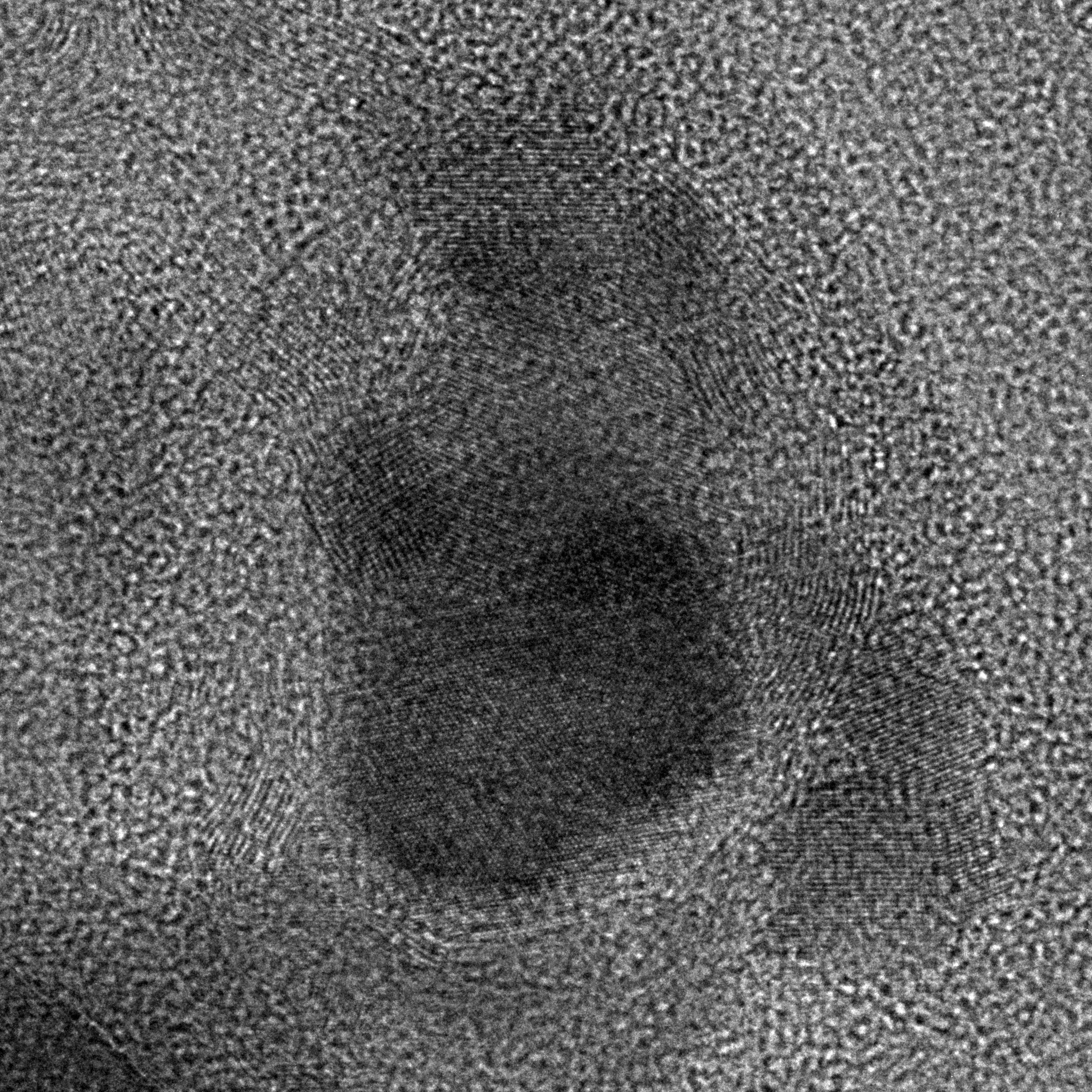

Supplement: Supplementary file 3 — Source Data [file 41467_2022_32024_MOESM3_ESM.zip › Source Data/SourceData1/FigS5c.jpg]

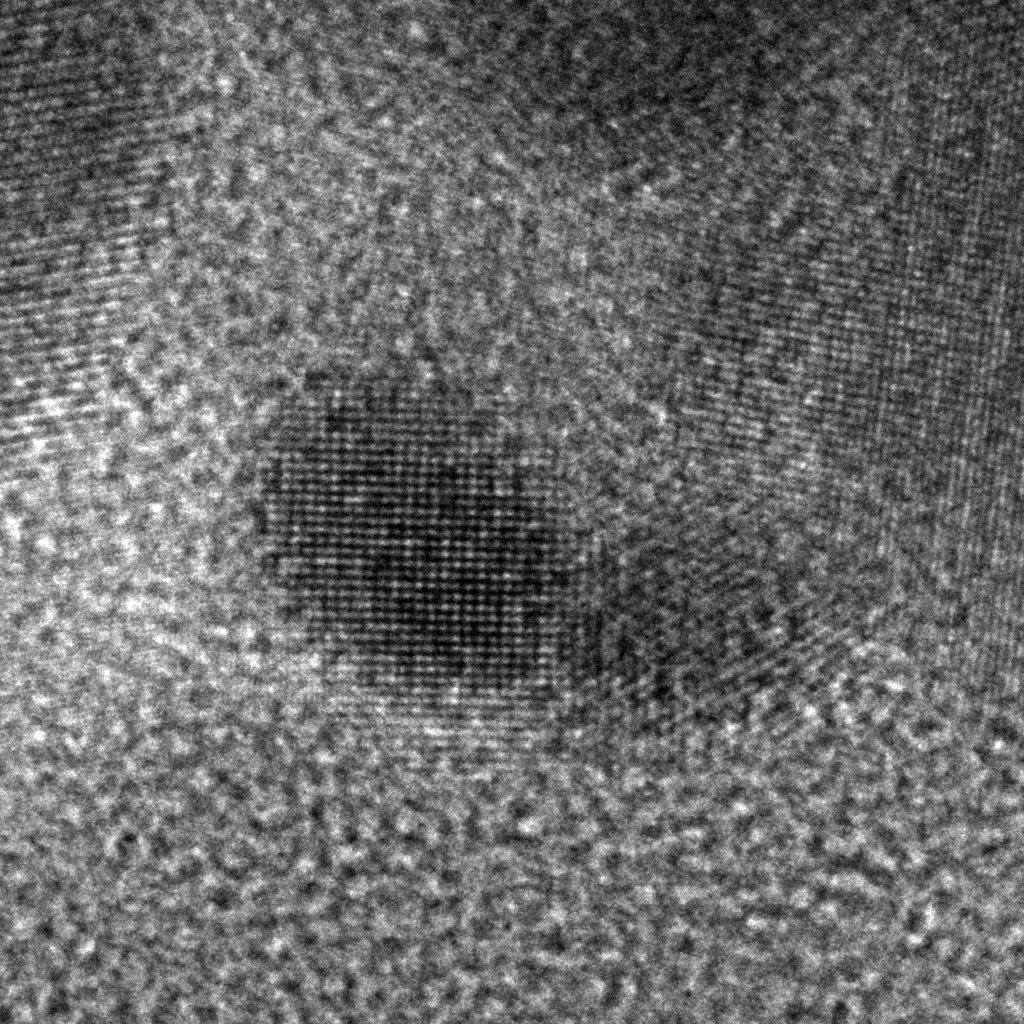

Supplement: Supplementary file 3 — Source Data [file 41467_2022_32024_MOESM3_ESM.zip › Source Data/SourceData1/FigS5b.jpg]

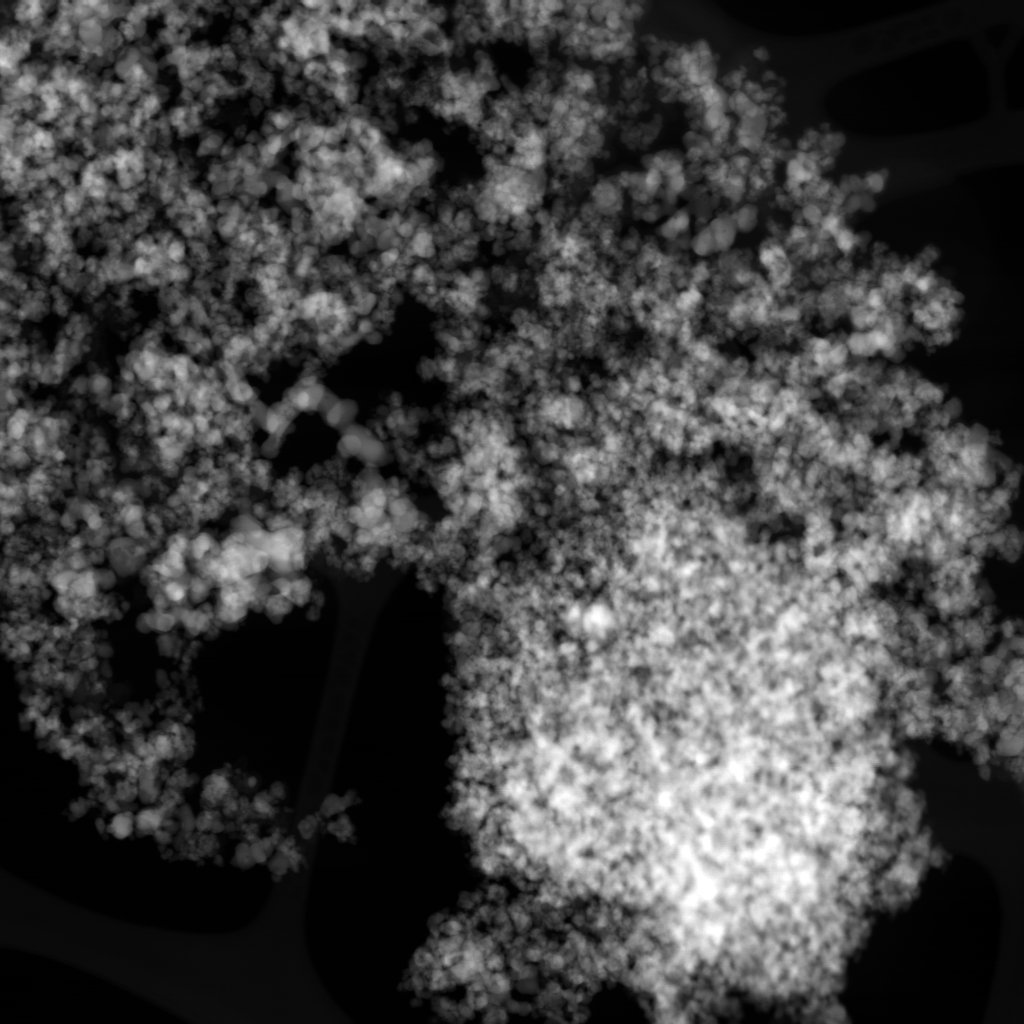

Supplement: Supplementary file 3 — Source Data [file 41467_2022_32024_MOESM3_ESM.zip › Source Data/SourceData1/FigS5a.jpg]

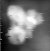

Supplement: Supplementary file 3 — Source Data [file 41467_2022_32024_MOESM3_ESM.zip › Source Data/SourceData1/FigS8_SI.jpg]

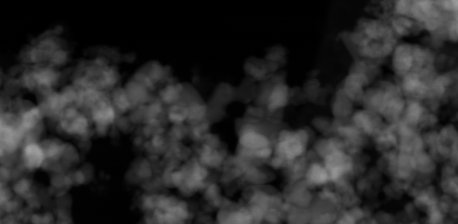

Supplement: Supplementary file 3 — Source Data [file 41467_2022_32024_MOESM3_ESM.zip › Source Data/SourceData1/FigS6_STEM.jpg]

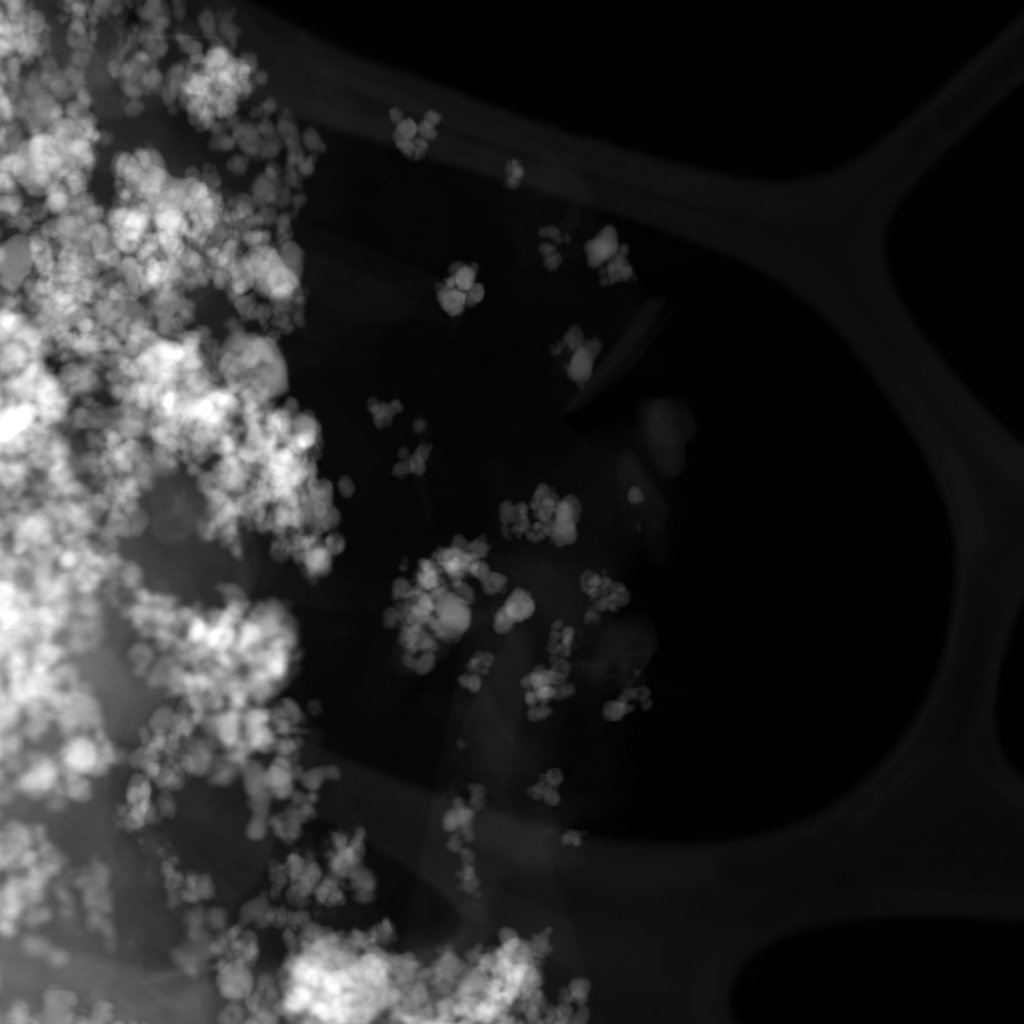

Supplement: Supplementary file 3 — Source Data [file 41467_2022_32024_MOESM3_ESM.zip › Source Data/SourceData1/Fig2a.jpg]

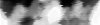

Supplement: Supplementary file 3 — Source Data [file 41467_2022_32024_MOESM3_ESM.zip › Source Data/SourceData1/FigS6_SI.jpg]

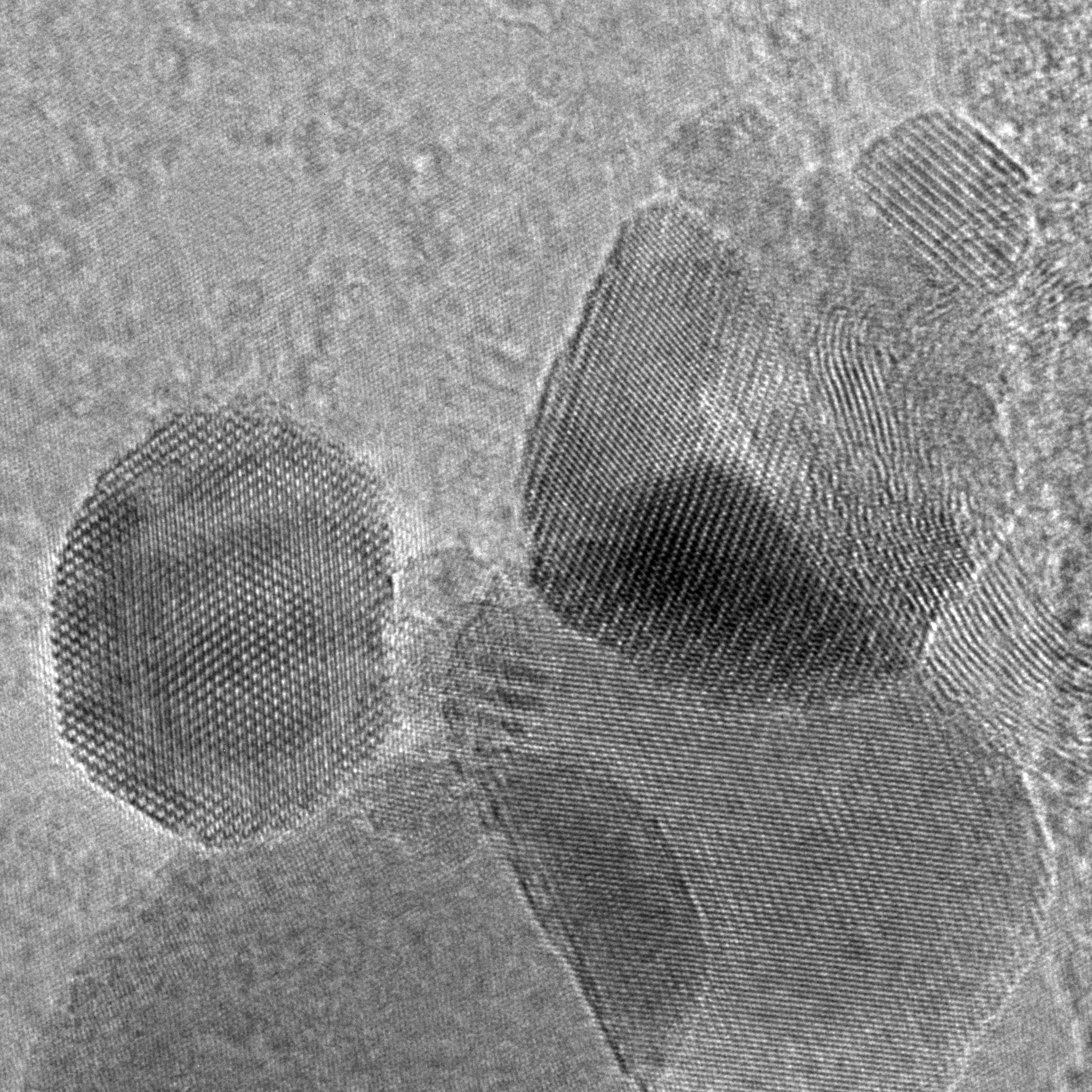

Supplement: Supplementary file 3 — Source Data [file 41467_2022_32024_MOESM3_ESM.zip › Source Data/SourceData1/Fig2b.jpg]
